# Modern Methods for the Sustainable Synthesis of Metalloporphyrins

**DOI:** 10.3390/molecules26216652

**Published:** 2021-11-02

**Authors:** Carla Gomes, Mariana Peixoto, Marta Pineiro

**Affiliations:** Department of Chemistry and CQC, University of Coimbra, Rua Larga 2, 3004-535 Coimbra, Portugal; carla_sofia.gomes@live.com.pt (C.G.); mariana.silva.fp@gmail.com (M.P.)

**Keywords:** metalloporphyrins, mechanochemistry, ultrasound, green chemistry, Atom Economy, E-factor, Ecoscale

## Abstract

Metalloporphyrins are involved in many and diverse applications that require the preparation of these compounds in an efficient manner, which nowadays, also involves taking into consideration sustainability issues. In this context, we use ball milling mechanochemistry and sonochemistry for the rational development of synthetic strategies for the sustainable preparation of metalloporphyrins. Zinc, copper, cobalt and palladium complexes of hydrophobic porphyrins were obtained in high yields and under mechanical action with a moderate excess of the metal salt, without any solvent or additive. Sonochemistry prove to be a good alternative for the preparation of metal complexes of water-soluble porphyrins in good yields and short reaction times. Both strategies have good sustainability scores, close to the ideal values, which is useful in comparing and helping to choose the more adequate method.

## 1. Introduction

**Figure 1:**
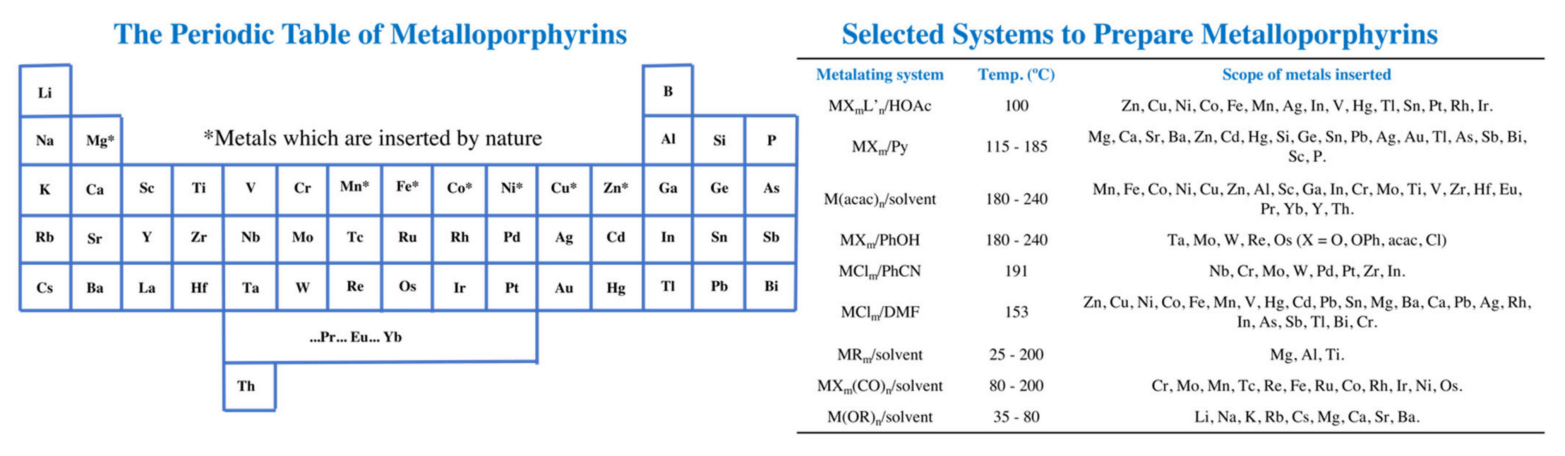
Periodic table of metalloporphyrins and metalating systems to prepare metalloporphyrins adapted from “The Porphyrins” Structure and Synthesis, PART A, Ed. D. Dolphin, Academic Press, New York, San Francisco, London 1978.

**Figure 2:**
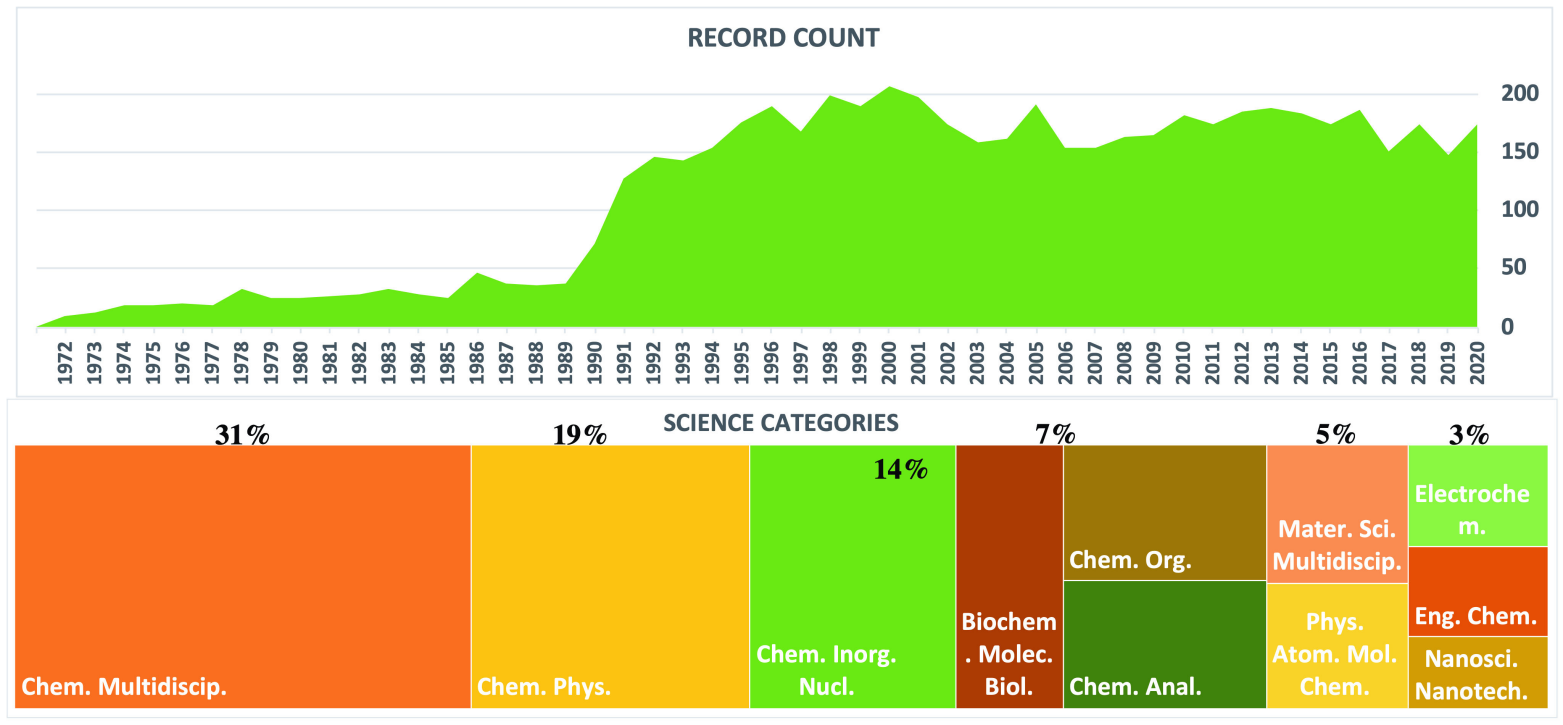
Results of the search in the Web of Science using metalloporphyrin as keyword in “all fields” (September 2021) presented as record count since 1972 and distribution by science categories (considering only the ones with more than 150 records).

The “pigments of life” are metal complexes of tetrapyrrolic macrocycles that play important roles in vital biological processes. Metal complexes of porphyrins and hydroporphyrins have been perfected by nature to give functional dyes for diverse applications. The most emblematic among them are involved in photosynthesis and in transport and activation of oxygen in several biological metabolisms. The role of the tetrapyrrolic macrocycle as a privileged ligand for the preparation of metal complexes was soon recognized, and in 1978, the periodic table of metalloporphyrins [1] (Figure 1) already included almost all the metals of the periodic table. Inspired by their roles in nature, the research involving metalloporphyrins is in constant development, the hundreds of papers/per year involving metalloporphyrins and their distribution among many science categories (Figure 2) illustrating very well the interest in these compounds and their applications in such diverse areas as catalysis, medicine, imaging, and materials [2,3,4,5,6,7,8,9,10,11,12,13,14,15,16]. 

**Chart 1:**
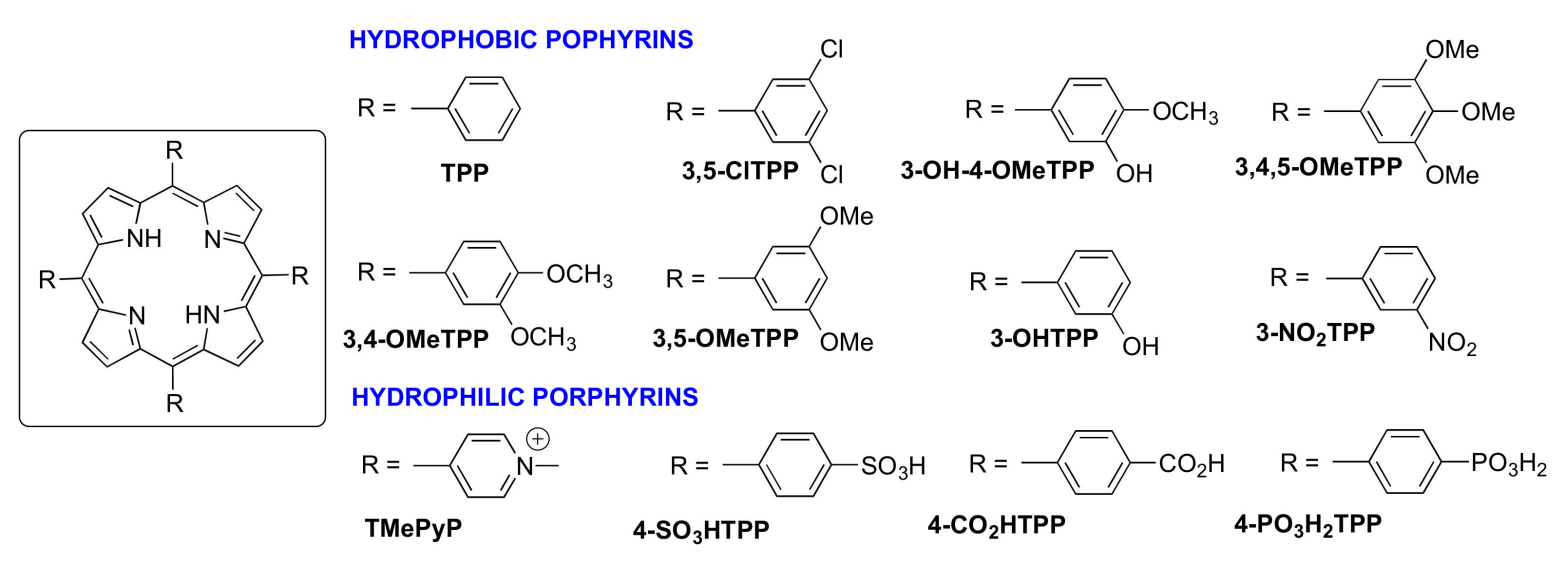
Porphyrins and acronyms used in this work.

The major difficulties of the synthesis of metalloporphyrins have been finding a reaction medium that allows the solubilization of both the metal salt and the organic compound, and forcing the equilibrium of the complexation process to form the product. Classical approaches solved the problem by using high boiling solvents (e.g., DMF) or mixtures of chlorinated solvents with alcohols (e.g., CHCl_3_/MeOH) and large excess of the metal salt (M(OAc)_2_; MCl_2_; etc) [1]. However, neither the use of those organic solvents nor the large excess of metal salts, which led to large quantities of chemical waste, are in line with current chemistry practices and present environmental concerns. Because the development of sustainable methods for the preparation of metalloporphyrins is essential to improve the sustainability of their applications in a bottom-up strategy, following the previously developed microwave assisted method [17], we focused on the exploration of mechanochemistry and ultrasound for the development of new methods for the preparation of metalloporphyrins of hydrophobic and hydrophilic porphyrins (Chart 1) looking for the increase in sustainability (quantified by Atom Economy, E-factor and Ecoscale). 

## 2. Results

### 2.1. Mechanochemistry for the Synthesis of Metalloporphyrins

The synthesis of metal complexes of *meso*-substituted porphyrins under mechanical activation was explored using 5,10,15,20-*tetrakis*-(3,4-dimethoxyphenyl)porphyrin (3,4-OMeTPP, Chart 1) as model compound, which was synthetized under microwave irradiation [17,18] or under mechanical action [19,20]. The scope of the reaction was evaluated with metals of the first transition row and group 10 of the periodic table. The reaction was followed by the UV-Vis of aliquots of the reaction crude dissolved in ethyl acetate. Optimization of the equivalent of the metal salt, milling frequency, reaction time and addition of NaOH allowed the synthesis of Zn(II), Cu(II), Co(III), Mn(III), Pd(II), and Pt(II) complexes in moderate to high yields (Table 1).

The Zn(II) complex was obtained quantitatively by milling 50 mg of porphyrin and 64 mg of Zn(OAc)_2_·2H_2_O (5 equivalent) in a stainless steel jar (10 mL) equipped with two stainless steel balls (7 mm of diameter) at 25 Hz for 30 min (entry 1, Table 1). The synthesis of the copper complex required the increase of the reaction time to 90 min to achieve 84% yield (entry 2, Table 1). The cobalt complex was obtained in good yield (61%) increasing the milling frequency to 30% and the reaction time to 250 min (entry 4, Table 1). The increase of the equivalent of salt and the reaction time only afforded 12% of the manganese (III) complex. However, the yield increased to a moderate 30% with the addition of 5 equivalent of NaOH (entry 6, Table 1). The preparation of the palladium (II) complex was achieved in good yield (70%) in the presence of NaOH and after 30 min of milling (entry 7, Table 1). The platinum complex was obtained with a moderate yield (50%) increasing the equivalent of NaOH to 10 (entry 9, Table 1). Iron and nickel complexes were not obtained under mechanical action (30 Hz during 300 min) when Fe(OAc)_3_, Fe(NO_3_)_3_ or Ni(OAc)_2_ and up to 10 equivalent of NaOH were used. In both cases the porphyrin was recovered without any detectable changes in the UV-Vis spectra. 

The scope of the reaction was increased using several *meso*-substituted porphyrins (Chart 1) and 5 equivalents of Cu(OAc)_2_·H_2_O under milling at 25 Hz (Table 2). The reaction was followed by UV-Vis and the milling was stopped when the Q bands of the porphyrin were not detectable. After liquid–liquid extraction with ethyl acetate/water to remove the excess of metal salt, all the copper complexes were obtained in very high yields in less than 90 min with little or no influence of the substituents of the phenyl ring (entries 1–7, Table 2). The only exception was the copper complex of TMePyP (entries 8 and 9, Table 2). After 90 min of milling TMePyP and 10 equivalents of Cu(OAc)_2_·H_2_O the Q band of the porphyrin free-base are not detectable however, after purification through molecular exclusion chromatography with Sephadex (G-10) only 35% yield was obtained. With the increase of the excess of salt (10 equivalents) or the reaction time (60 and 90 min) only 46% yield was achieved (entry 9 and 10, Table 1). 

### 2.2. Ultrasound for the Synthesis of Metalloporphyrins

**Figure 3:**
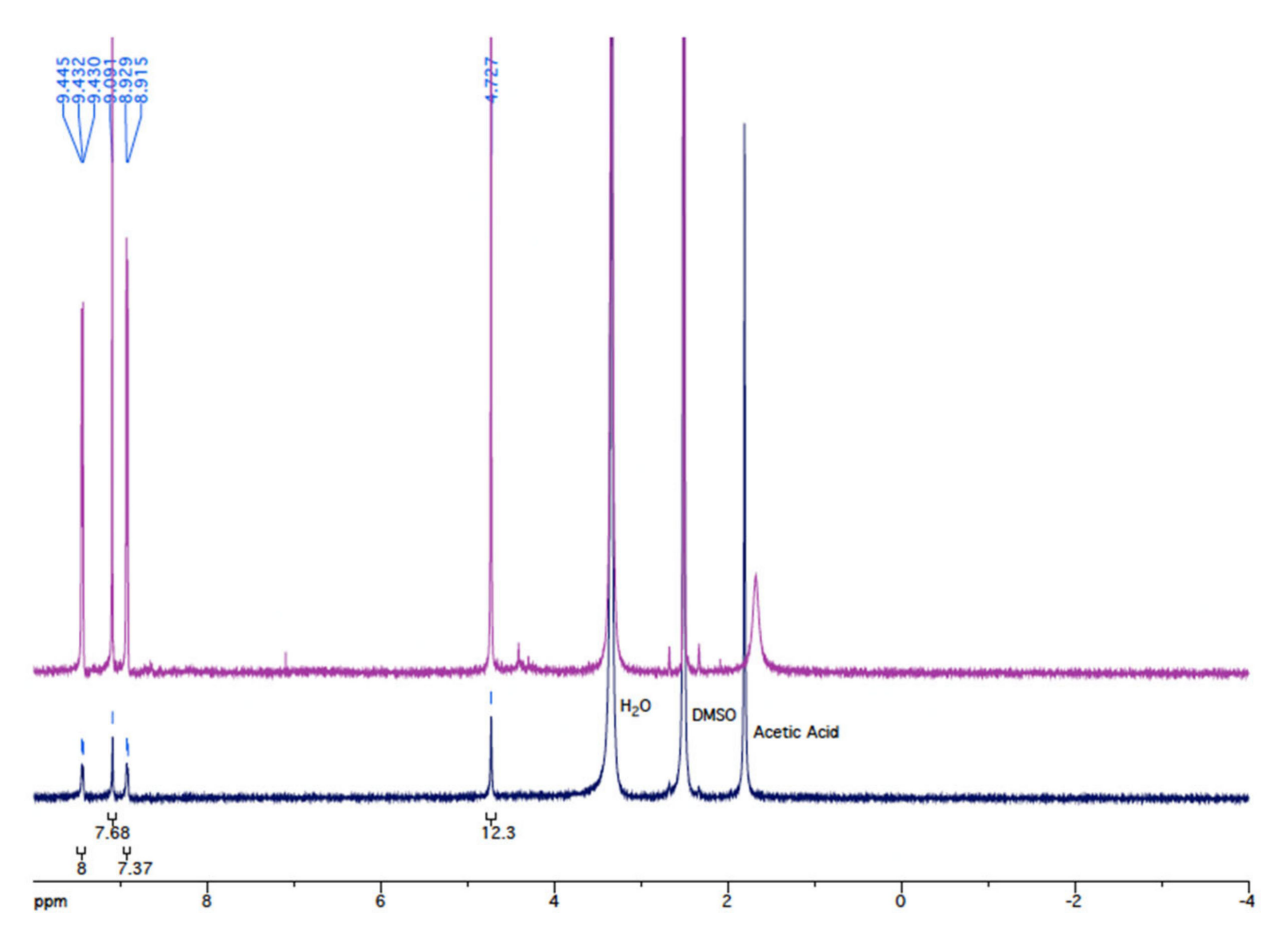
^1^H-NMR (DMSO-d_6_) of the reaction (TMePyP and 1 equivalent of Zn(OAc)_2_, 1 mL of water, 30 min under ultrasound) after solvent evaporation (blue line) and after purification through exclusion column chromatography (magenta line).

Looking for a method to synthesize water-soluble metalloporphyrins efficiently, we focused on the synthesis of metalloporphyrins under ultrasound. To be able to follow the reaction by ^1^H-NMR- we chose the synthesis of Zn(II)-5,10,15,20-*tetrakis*-*N*-methyl-4-pyridylporphyrinate as model reaction. This compound was synthesized with a moderate 50% yield under mechanical action after 60 min. The reaction conditions were optimize using as starting point 5 equivalents of metal salt and 1 mL of water, considered “The Green Solvent” in solvents’ guides [21]. After 30 min under ultrasound the Q bands of the porphyrin were undetectable by UV-Vis indicating quantitative complexation. To decrease the reaction waste, the amount of metal salt was reduced to 1 equivalent after 30 min the free porphyrin was undetectable by UV-Vis, the solvent was evaporated and the reaction crude analyzed by ^1^H-NMR (Figure 3). The inner protons of the free porphyrin were undetectable by NMR while the hydrogens of the acetate from the metal salt ligand were observable. After purification through exclusion chromatography a notable reduction of the acetate peak was observed in the ^1^H-NMR spectrum (Figure 3) and 90% yield was obtained (entry 1, Table 3).

**Figure 4:**
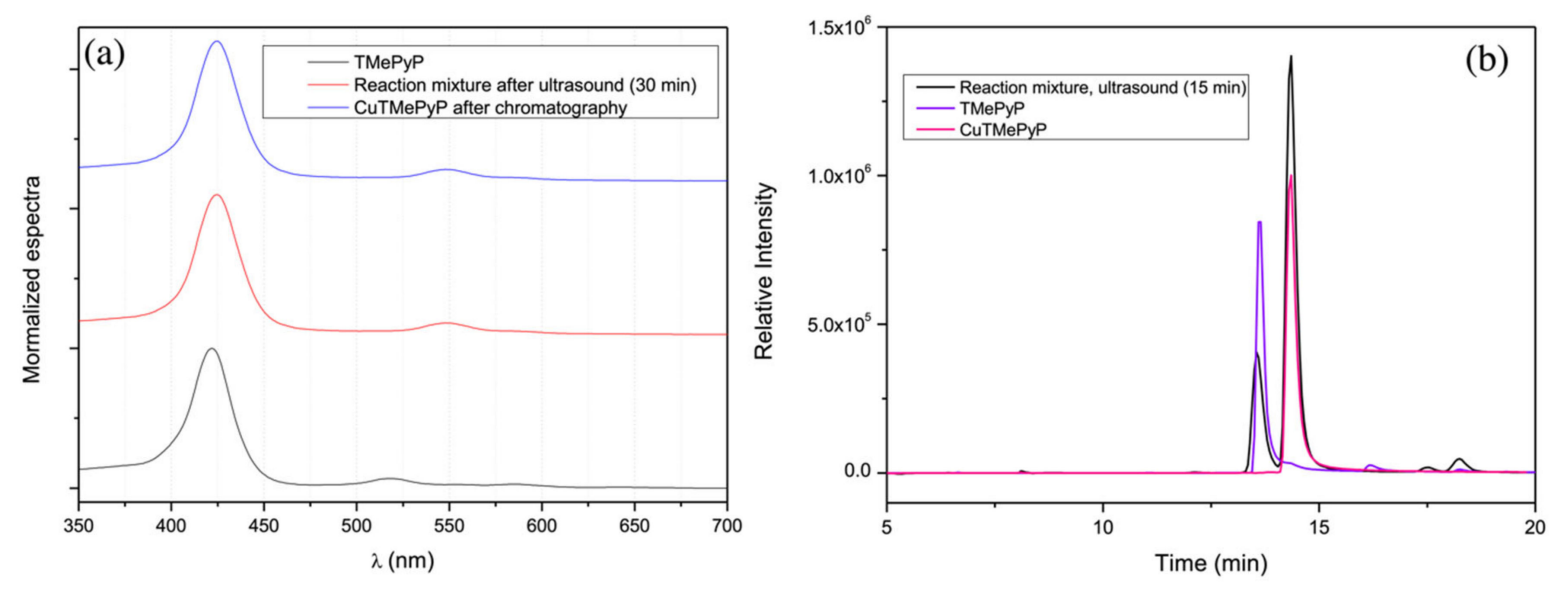
(**a**) Normalized UV-Vis spectra of free porphyrin, TMePyP (black), the reaction crude after 30 min under ultrasound -TMePyP and 1 equivalent of Cu(OAc)_2_·H_2_O in 1 mL of water-(red) and the copper complex CuTMePyP after purification through exclusion chromatography (blue) (**b**) HPLC chromatograms of the reaction crude -TMePyP and 1 equivalent of Cu(OAc)_2_·H_2_O in 1 mL of water after 30 min under ultrasound (black) compared with the free porphyrin TMePyP (blue) and the copper complex CuTMePyP after isolation and purification through exclusion chromatography (pink).

The same reaction conditions were tested for the preparation of the copper (II) complex. After 30 min under ultrasound the free porphyrin was not observable by UV-Vis (Figure 4a). However, the HPLC analysis of the reaction crude shows the presence of 21% of free porphyrin (Figure 4b). After exclusion chromatography purification, 53% yield of copper complex was obtained (entry 2, Table 3). The above described reaction conditions were applied for the complexation of 200 mg of 5,10,15,20-*tetrakis*-*N*-methyl-4-pyridylporphyrin in 1 mL of water with Zn(OAc)_2_·2H_2_O and Cu(OAc)_2_·H_2_O yielding 57% and 37% of the corresponding metal complexes. 

The manganese (III) complex was obtained in 79% yield after 180 min under ultrasound (entry 3, Table 3). The nickel and cobalt complexes were obtained in moderated isolated yields (entry 4 and 5 at Table 3, respectively). The complexation of water-soluble porphyrins with sulfonic, carboxylic and phosphonic acid substituents (entries 1–5, Table 4) was achieved in high yields with 1 equivalent of Zn(OAc)_2_·2H_2_O or Cu(OAc)_2_·H_2_O in 1 mL of NaOH (2 M) under ultrasound irradiation. In the case of the copper complex of 5,10,15,20-*tetrakis*-(4-carboxyphenyl)porphyrin quantitative isolated yield was obtained using 2 equivalents of the copper salt (entry 4, Table 4). The same reaction conditions were used for the synthesis of less hydrophilic 5,10,15,20-*tetrakis*-(3-hydroxyphenyl)porphyrin in 89% yield.

### 2.3. Sustainability Assesment

To evaluate the sustainability of the developed synthetic processes using mechanochemistry and ultrasound and to compare them Atom Economy [22], E-factor [23] and Ecoscale [24] values were determined for the synthesis of TMePyP and 3-OHTPP using both strategies. The data is presented in Table 5. For details see Tables S1–S5 at Supplementary Materials.

Atom Economy, the number of atoms of reactants appearing in the products (Equation 1), is a measurement of the reaction efficiency complementary to the reaction yield:

% Atom Economy = (MW of the product/sum of the MW of the reactants) × 100(1)

The E-factor is the ratio of the mass of waste per mass of product (Equation 2) taking into consideration reactants, solvents and reaction auxiliaries:

E-factor = (total mass of waste/mass of product)(2)

Both in the case of Atom Economy and E-factor, the lower the value, the more sustainable can be considered the process. The Ecoscale is a post-synthetic tool that evaluates other aspects of the synthetic process, namely, toxicity of the reactants, energy input (time and temperature of the reaction) and procedure safety (see Table S5 in Supplementary Materials). All the procedures to be analyzed start with 100 points, and points are subtracted as each of the evaluated parameters deviates from the ideal of sustainability. Therefore, in the case of Ecoscale, the higher the value, the more sustainable the process will be.

## 3. Discussion

Milling of *meso*-substituted porphyrins (with electron withdrawing and donor substituents) and five equivalents of zinc, copper and cobalt acetate allows the efficient synthesis of the corresponding metalloporphyrins in less than 4 h and without the need of any additional solvent or additive (Table 1 and Table 2). The purification to remove the excess of metal salt is efficiently done by simple liquid–liquid extraction using solvents with good sustainability scores, such as ethyl acetate and water. The use of the excess of metal salt has a negative effect on the Atom Economy. However, the solvent-free, additive-free strategy paves the way to an impressive E-factor value of 2.1 (entry 3, Table 5), very close to the ideal value (zero waste), which is also corroborated by a Ecoscale value of 72 points, where the deviation from the ideal value (100 points) is due to the inherent cost of the porphyrin and the toxicity of the metal salt (entry 3, Table 5 and Table S5 at Supplementary Materials). 

The synthesis of manganese, palladium and platinum metalloporphyrins is more challenging [25,26]. The synthesis of these compounds in moderated yields required the use of a larger excess of the corresponding chloride salt, the addition of NaOH to neutralize the hydrogen chloride formed in situ and long reaction times (Table 1).

The solvent-free mechanochemical synthesis of the copper complex of the water-soluble TMePyP was achieved in moderate yield, Table 2. The UV-Vis spectrum after 60 min of milling at 30 Hz shows the Q band at 549 nm, characteristic of the metalloporphyrin in water, while the Q bands of the free porphyrin are undetectable. However, the purification of the metalloporphyrin requires an exclusion column chromatography, to remove the excess of metal salt and acetic acid formed in situ, after which only 45% of the metalloporphyrin was recovered. Even considering this purification process the sustainability scores (E-factor = 4.7 and Ecoscale = 50.5 points) reflect well the greenness of the mechanochemical process (entry 1, Table 5).

**Figure 5:**
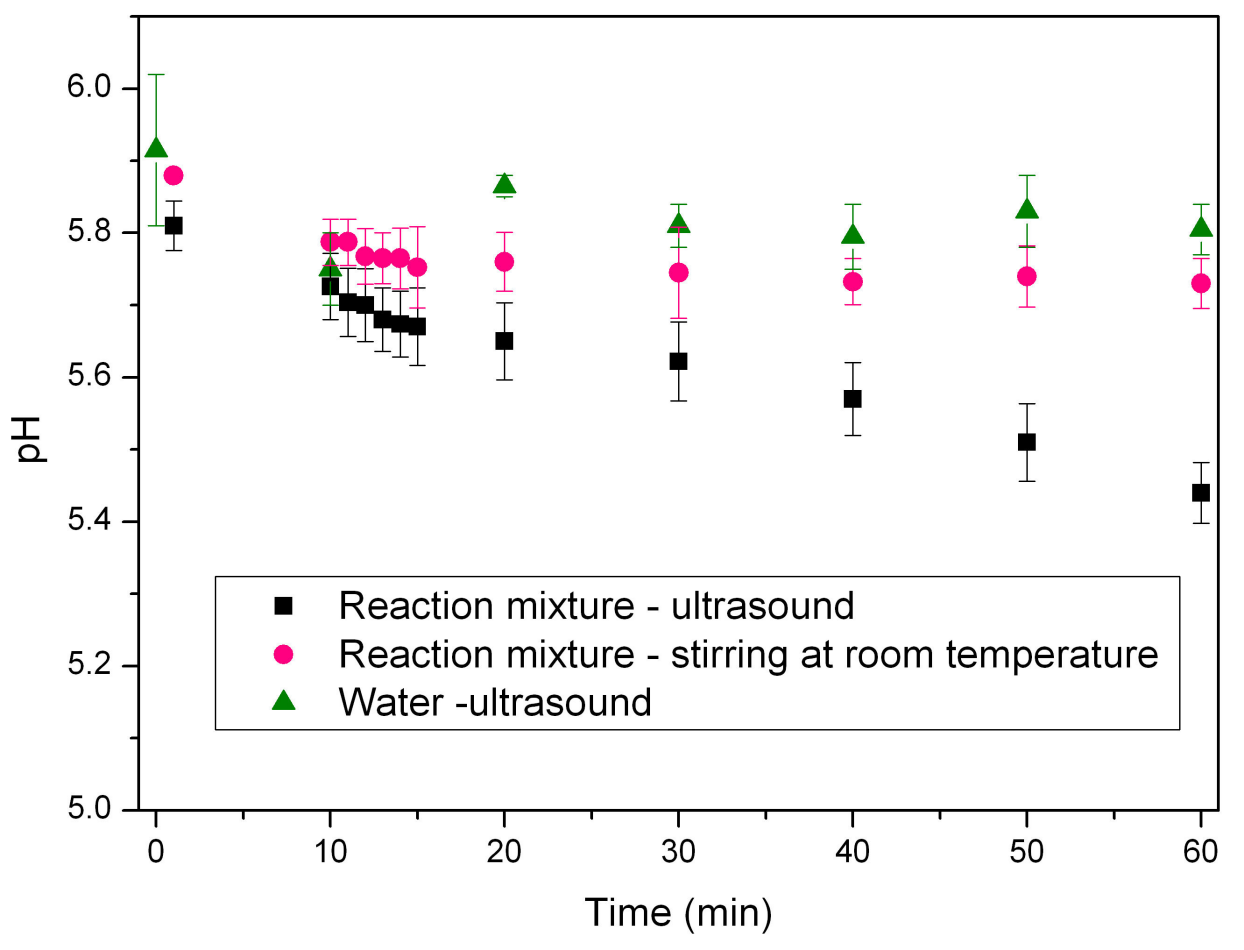
pH of the metalation reaction (TMePyP and 1 equivalent of Cu(OAc)_2_·H_2_O in 1 mL of water) vs. time under ultrasound (5 independent experiments, black) and under stirring at room temperature (2 independent experiments, pink), pH of water under ultrasound (2 independent experiments green).

**Scheme 1:**
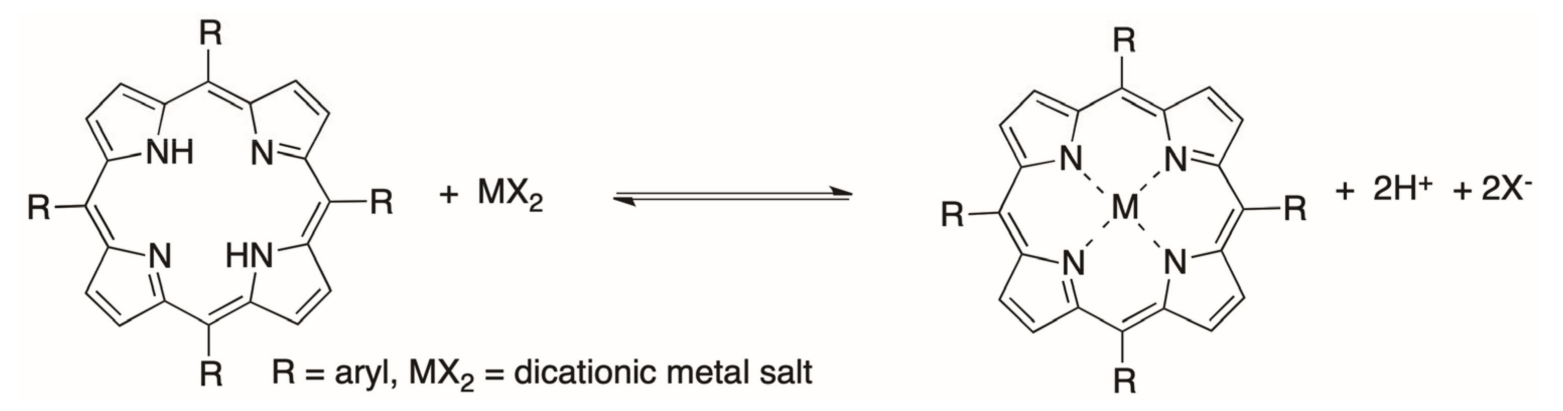
Synthesis of metalloporphyrins.

Looking for a way to reduce the excess of metal salt and since the porphyrin is water-soluble the use of ultrasound as activation technique was tested. The Zn(II) complex was obtained in 90% yield from the free porphyrin with just 1 equivalent of the metal salt in 1 mL of water after 30 min under ultrasound, Table 3. The copper complex was obtained in 79% yield (determined by HPLC) and 53% isolated yield. Monitoring the pH of the metalation reaction under ultrasound and comparing with the same reaction under stirring at room temperature, it is possible to observe a slight decrease under ultrasound irradiation (Figure 5). This could be associated with the mechanism proposed for the metalation process, involving the removal of the inner protons of the porphyrin core, Scheme 1, and could be responsible for the low yields obtained with more challenging metals. 

Despite the mass lost in the chromatographic process, the synthesis of metalloporphyrins of TMePyP under ultrasound is effective and has very relevant sustainability scores (entry 2, Table 5), The Atom Economy dramatically increases due to the use of the stoichiometric amount of metal salt, reaching 90% the highest value possible for this reaction due to the inherent elimination of acetic acid. The use of water has a negative effect on the E-factor because is considered mass of solvent added to the process; however, it does not modify the Ecoscale (54.5 points) because it is a cheap and nontoxic solvent. 

**Figure 6:**
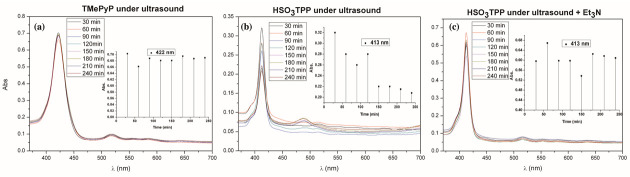
UV-Vis spectra of (**a**) TMePyP and (**b**) 4-SO_3_HTPP in water under ultrasound irradiation for 240 min. (c) UV-Vis spectra of 4-SO_3_HTPP in water under ultrasound irradiation for 240 min measured after the addition of 1 drop of Et_3_N. Inset: Absorption at the Soret band maximum vs. time.

To increase the reaction scope water-soluble porphyrins with sulfonic, carboxylic and phosphonic acid substituents were tested. Tetrasulfonic porphyrin (4-SO_3_HTPP) was previously report as a sensor for quantification of ultrasound intensity based on the decrease of the Soret band absorption observed under sonification [27,28]. Comparing the evolution of the UV-Vis spectra with the sonification time of a solution of TMePyP and 4-SO_3_HTPP, the different behavior is clear: while TMePyP is unchanged over the time the absorption maximum of the Soret band of 4-SO_3_HTPP decreases under ultrasound irradiation. Interestingly, with the addition of triethylamine the absorption of the Soret recovers to its original value, indicating that the decreases of the absorption could be related to the change in pH of the porphyrin solution under ultrasound (Figure 6). Considering that the metalation is an equilibrium involving the removal of the inner protons of the porphyrin, Scheme 1, the metalation of the water-soluble porphyrins bearing acidic substituents was performed using 1 mL of aqueous NaOH solution (2 M) as solvent.

The Zn(II) complexes of tetrasubstituted sulphonic, carboxylic and phosphonic phenylporphyrins were synthetized using Zn(OAc)_2_·2H_2_O in 1:1 molar ratio in 1 mL of NaOH aqueous solution (2 M) under ultrasound irradiation for less than 90 min, with moderate to good yields (entries 1, 2 and 5, Table 4). The reaction conditions were also applied for the synthesis of the Cu(II) complex of the carboxylated porphyrin, which was obtained in 85% yield (entry 3, Table 4) when 1 equivalent of copper acetate was used and in 99% using two equivalent of the metal salt (entry 4, Table 4).

The use of NaOH solution as solvent opens the way to the metalation of less hydrophilic porphyrins under ultrasound. This is the case of 3-OHTPP, which solubilizes under these conditions allowing the preparation of its Zn(II) complex with 89% yield. In this case the neutralization of the reaction allows the isolation and purification of the metal complex by liquid–liquid extraction using ethyl acetate as solvent. Comparing with the use of mechanochemistry for the preparation of this complex (entry 3, Table 5), ultrasound allows the use of the metal salt in 1:1 molar ratio, increasing the Atom Economy to almost ideal values. However, the use of a solvent increases the value of the E-factor to 28.3, shifting it far from the ideal value. The Ecoscale, also moves beyond the ideal value due to the use of NaOH (entry 4, Table 5).

## 4. Materials and Methods

### 4.1. Reactants and Equipment

All commercially acquired reagents were high-grade chemicals and were used without any additional purification: Metal salts- Zinc acetate dihydrate (5970-45-6, *Merck*, 99%), Copper(II) acetate monohydrate (142-71-2; *Baker Analyzed Reagent*), Cobalt(II) acetate tetrahydrate (6147-53-1, *Merck*, 99%), Manganese(II) acetate tetrahydrate (6156-78-1, *Riedel-de* *Haën*, 95–97%), Palladium(II) chloride (7647-10-1, *Fluorochem*, 99%) and Platinum(II) chloride (10025-65-7, *Fluorochem*, 99%)- Hydrophilic porphyrins- 5,10,15,20-(tetra-*N*-methyl-4-pyridyl)porphyrin tetraiodide (36674-90-5, *PorphyChem*, >98%, *Lot* 257ABo19), 5,10,15,20-(tetra-4-sulfonatophenyl)porphyrin tetraamonium (39174-47-5, *PorphyChem*, >95%, *Lot* 047ESi19), 5,10,15,20-(tetra-4-carboxyphenyl)porphyrin (14609-54-2, *PorphyChem*, >98%, *Lot* 11ESi19) and 5,10,15,20-(tetra-4-phosphonatophenyl)porphyrin (143969-69-1, *PorphyChem*, >95%, *Lot* 102ESi16). All commercially acquired solvents were purified according to literature procedures prior to use [29].

Ball milling reactions were performed in a Retsch MM 400 with constant frequency and time monitoring. Stainless steel jars (10 mL) and stainless-steel spheres (7 mm diameter) were used. HPLC was carried out using Elite Lachrom HPLC-DAD system with L-2455 Diode Array Detector, L-23000 Column Oven (RP-18 endcapped column-((5 μm) from Merck), L-2130 Pump and a L-2200 Auto Sampler. Reactions under ultrasound were performed in a Bandelin Sonorex RK100H. NMR spectra were registered at room temperature (RT) in a Bruker Avance III spectrometer, operating at 400 MHz. TMS was the internal standard used. Chemical shifts (δ) and coupling constants (J) are indicated in ppm and Hz, respectively. UV-Vis absorption spectra were obtained on a *pg instruments T80 UV/VIS* Spectrometer. pH was measured using a Microelectrode 3510-pHmeter jenway.

### 4.2. HPLC Analysis Method

HPLC-DAD analysis using as stationary phase Purospher^®^ STAR RP-18 endcapped (5 μm) and as eluent: potassium acetate (pH = 3)(A) and MeCN:H_2_O (50:50)(B) (from 100:0 to 30:70 (A/B *v*/*v*)); 5% increment of B each min. for 12 min; 5% increment of B each 2 min. until 22 min and 30:70 (A/B *v*/*v*) ratio for 12 min. Flow rate of 0.8 mL/min in the first 9 min and then 0.4 mL/min until 35 min. L-2455 Diode Array Detector (400 nm). TMePyP: Percentage of total chromatogram integration at retention time 13.7 min relative area 98%. Cu(II)-TMePyP after purification: Percentage of total chromatogram integration at retention time 14.2 min relative area 98%.

### 4.3. General Mechanochemical Synthesis Metalloporphyrins

A mixture of the porphyrin (50 mg), the metal salt (5 equivalent) and two stainless steel spheres (7 mm of diameter) were placed in the stainless-steel jar and submitted to mechanical action in a ball milling system (Retsch 400 MM) at 25 Hz during 30–90 min. The reaction was followed by UV-Vis at each 30 min. Once complete, the reaction product was scraped to empty the jar. Hydrophobic porphyrins were purified through liquid–liquid extraction using ethyl acetate and water, the organic phase was dried with Na_2_SO_4_ anhydride and the solvent removed through evaporation. Water-soluble porphyrins were purified through exclusion chromatography (Sephadex G-10) using water as eluent, followed by water evaporation.

For details on the characterization of the synthesized metalloporphyrins see Supplementary Materials.

### 4.4. General Synthesis of Metalloporphyrins under Ultrasound

A mixture of the porphyrin (50 mg), the metal salt (1 equivalent) and 1 mL of water or NaOH (2 M) aqueous solution were placed in a glass container (microwave reactor glass vial-10 mL) and placed into the ultrasound bath (Bandelin Sonorex RK100H) ensuring that all the reaction solution is below the level of the water at the ultrasound bath (ca. 1 cm depth). The reaction was followed by UV-Vis at each 10 min. Once complete, the reaction was neutralized with HCl (1 M) solution. The hydrophobic porphyrins were purified through liquid–liquid extraction using ethyl acetate, the organic phase was dried with Na_2_SO_4_ anhydride and the solvent removed through evaporation. Water-soluble porphyrins were purified through exclusion chromatography (Sephadex G-10) using water as eluent, followed by water evaporation.

For details on the characterization of the synthesized metalloporphyrins see Supplementary Materials.

## 5. Conclusions

The synthesis of metal complexes of *meso*-substituted porphyrins bearing hydrophobic and hydrophilic substituents was achieves in good yields and with good sustainability scores under mechanical action or ultrasound irradiation. The use of ball milling mechanochemistry allows the solvent-free synthesis of zinc, copper, cobalt and palladium complexes of hydrophobic porphyrins with a moderate excess of the metal salt, without the need of any additional additive. The synthesis of metal complexes of hydrophilic porphyrins under mechanochemical action require the use of excesses of metal salts and the addition of NaOH to achieve moderate yields. Sonochemistry is a good alternative for the preparation of metal complexes of water-soluble porphyrins. Under ultrasound irradiation using water as solvent and a stoichiometric amount the zinc, copper and manganese acetate the complexes of 5,10,15,20-*tetrakis*-*N*-methyl-4-pyridylporphyrin were obtained in excellent yields. The complexation of water-soluble porphyrins bearing ionizable groups in aqueous basic solutions (hydroxy, sulfonic, carboxylic and phosphonic substituents) requires the use of NaOH aqueous solution but could be achieved without the use of an excess of the metal salt, in good yields and short reaction times.

From the sustainability point of view, the use of an excess of metal salt, needed to efficiently perform the reaction under mechanochemical action has a great impact on the Atom Economy, shifting this metric from the ideal value almost achieved under ultrasound irradiation. The E-factor is more sensitive to the use of solvent, even a green solvent such as water, since only the mass of solvent is taken into consideration and not its characteristics and, therefore the synthesis under ultrasound irradiation compares unfavorable with the use of mechanochemistry. Looking to a more comprehensive metric, the Ecoscale, which also considers the reaction conditions and purification techniques, it could be inferred that neither the use of water as solvent nor the increase of the equivalent of metal salt have a negative impact on sustainability. The addition of an additive (NaOH) has a negative impact mostly due to the hazard considerations and, above all the chromatography-free strategies have very high sustainability scores.

In sum, metalloporphyrins can be prepared in an efficient and sustainable way using mechanical action and/or under ultrasound irradiation. The strategy to use should be chosen based on the hidrophylicity/hydrophobicity of the porphyrin and on the sustainability metrics, which furnish the basis for a conscious choice.

## Figures and Tables

**Table 1:** Synthesis of metal complexes of 5,10,15,20-*tetrakis*-(3,4-methoxyphenyl)porphyrin, 3,4-OMeTPP, under mechanical action.

Entry	Metal Salt	Equiv	NaOH (Equiv)	Milling Freq (Hz)	t(min)	Yield ^1^ (%)
1	Zn(OAc)_2_·2H_2_O	5		25	30	97
2	Cu(OAc)_2_·H_2_O	5		25	90	84
3	Co(OAc)_2_·4H_2_O	5		25	150	46
4	Co(OAc)_2_·4H_2_O	5		30	250	61
5	Mn(OAc)_2_·4H_2_O	10		30	300	12
6	Mn(OAc)_2_·4H_2_O	10	5	30	300	30
7	PdCl_2_	10	5	30	30	70
8	PtCl_2_	10	5	30	150	8
9	PtCl_2_	10	10	30	300	50 ^2^

^1^ Isolated yields obtained after liquid–liquid extraction with ethyl acetate/water to remove the excess of metal salt. ^2^ Calculated by ^1^H-NMR.

**Table 2:** Synthesis of copper complexes of *meso*-substituted porphyrins under mechanical action (milling frequency 30 Hz).

Entry	Porphyrin	Cu(OAc)_2_·H_2_O Equiv	t(min)	Yield ^1^ (%)
1	3,4-OMeTPP	5	90	84
2	TPP	5	90	75
3	3,5-OMeTPP	5	30	89
4	3,4,5-OMeTPP	5	30	88
5	3-OH-4-OMeTPP	5	30	90
6	3,5-ClTPP	5	45	97
7	3-OHTPP	5	30	74
7	3-NO_2_TPP	5	30	70
8	TMePyP	5	30	35 ^2^
9	TMePyP	10	60	45 ^2^
10	TMePyP	10	90	46 ^2^

^1^ Isolated yields obtained after liquid–liquid extraction with ethyl acetate/water to remove the excess of metal salt. ^2^ After purification (Exclusion chromatography-Sephadex).

**Table 3:** Synthesis of metal complexes of 5,10,15,20-*tetrakis*-*N*-methyl-4-pyridylporphyrin under ultrasound.

Entry	Metal Salt	Equiv	t(min)	Yield ^1^ (%)
1	Zn(OAc)_2_·2H_2_O	1	30	90
2	Cu(OAc)_2_·H_2_O	1	30	53 (79) ^2^
3	Mn(OAc)_2_·4H_2_O	1	180	79
4	Ni(OAc)_2_·4H_2_O	5	330	18
5	Co(OAc)_2_·4H_2_O	2	120	32

^1^ Isolated yields obtained after exclusion chromatography. ^2^ Yield by HPLC.

**Table 4:** Synthesis of zinc and copper complexes of water-soluble porphyrins under ultrasound irradiation in 1 mL of NaOH (2 M).

Entry	Porphyrin	Metal Salt	Equiv	t(min)	Yield ^1^ (%)
1	4-SO_3_HTPP	Zn(OAc)_2_·2H_2_O	1	60	65
2	4-CO_2_HTPP	Zn(OAc)_2_·2H_2_O	1	30	46
3	4-CO_2_HTPP	Cu(OAc)_2_·H_2_O	1	60	85
4	4-CO_2_HTPP	Cu(OAc)_2_·H_2_O	2	60	99
5	4-PO_3_H_2_TPP	Zn(OAc)_2_·2H_2_O	1	90	32
6	3-OHTPP	Zn(OAc)_2_·2H_2_O	1	30	89 ^2^

^1^ Isolated yields obtained after exclusion chromatography (Sephadex) using water as eluent to remove the excess of metal salt. ^2^ Isolated yields after purification through liquid–liquid extraction with ethyl acetate after neutralization with HCl solution.

**Table 5:** Sustainability parameters.

	Porphyrin	Method	Cu(OAc)_2_·H_2_O Equiv	Solvent	T (min)	Yield ^1^ (%)	Atom Economy	E-Factor	Ecoscale
1	TMePyP	Mechanochemistry	10		60	45	57%	4.7	50.5
2	TMePyP	Ultrasound	1	1 mL H_2_O	30	53	90%	37.0	54.5
3	3-OHTPP	Mechanochemistry	5		30	74	44%	2.1	72
4	3-OHTPP	Ultrasound	1	1 mL NaOH (2 M)	30	74	84%	27.3	67

^1^ Isolated yields.

## Data Availability

Not applicable.

## Supplementary Materials

The following are available online, Figure S1. UV-Vis and ^1^H-NMR spectra of Zn(II) 5,10,15,20-*tetrakis*-(3,4-dimethoxyphenyl)porphyrinate, Figure S2. UV-Vis of Cu(II) 5,10,15,20-*tetrakis*-(*N*-methyl-4-pyridinyl)porphyrinate tetraiodide. Figure S3. UV-Vis and HRMS of Cu(II) 5,10,15,20-*tetrakis*-(3,4-dimethoxyphenyl)porphyrinate. Figure S4. UV-Vis of Co(III) 5,10,15,20-*tetrakis*-(3,4-dimethoxyphenyl)porphyrinate acetate. Figure S5. UV-Vis and HRMS spectra of Mn(III) 5,10,15,20-(3,4-dimethoxyphenyl)porphyrinate acetate. Figure S6. UV-Vis and ^1^H-NMR spectra of Pd(II) 5,10,15,20-*tetrakis*-(3,4-dimethoxyphenyl)porphyrinate. Figure S7. UV-Vis and ^1^H-NMR spectra of Pt(II) 5,10,15,20-*tetrakis*-(3,4-dimethoxyphenyl)porphyrinate. Figure S8. UV-Vis and HRMS of Cu(II) 5,10,15,20-tetraphenylporphyrinate. Figure S9. UV-Vis and HRMS of Cu(II) 5,10,15,20-*tetrakis*-(3,5-dimethoxyphenyl)porphyrinate. Figure S10. UV-Vis and HRMS of Cu(II) 5,10,15,20-*tetrakis*-(3,4,5-trimethoxyphenyl)porphyrinate. Figure S11. UV-Vis of Cu(II) 5,10,15,20-*tetrakis*-(3-hydroxyphenyl)porphyrinate. Figure S12. UV-Vis and HRMS of Cu(II) 5,10,15,20-*tetrakis*-(3-hydroxyphenyl-4-methoxyphenyl))porphyrinate. Figure S13. UV-Vis and HRMS of Cu(II) 5,10,15,20-*tetrakis*-(3,5-dichlorophenyl)porphyrinate. Figure S14. UV-Vis and of Cu(II) 5,10,15,20-*tetrakis*-(3-nitrophenyl)porphyrinate. Figure S15. UV-Vis and ^1^H-NMR of Zn(II) 5,10,15,20-*tetrakis*-(*N*-methyl-4-pyridinyl)porphyrinate tetraiodide. Figure S16. UV-Vis of Mn(III) 5,10,15,20-*tetrakis*-(*N*-methyl-4-pyridinyl)porphyrinate tetraiodide. Figure S17. UV-Vis of Ni(II) 5,10,15,20-*tetrakis*-(*N*-methyl-4-pyridinyl)porphyrinate tetraiodide. Figure S18. UV-Vis of Co(III) 5,10,15,20-*tetrakis*-(*N*-methyl-4-pyridinyl)porphyrinate tetraiodide. Figure S19. UV-Vis and ^1^H-NMR of Zn(II) 5,10,15,20-*tetrakis*-(4-phosphonatophenyl)porphyrinate. Figure S20. UV-Vis and ^1^H-NMR of Zn(II) 5,10,15,20-*tetrakis*-(4-carboxyphenyl)porphyrinate. Figure S21. UV-Vis and ^1^H-NMR of Zn(II) 5,10,15,20-*tetrakis*-(3-hydroxyphenyl)porphyrinate. Figure S22. UV-Vis and ^1^H-NMR of Zn(II) 5,10,15,20-*tetrakis*-(4-sulfonatophenyl)porphyrinato tetraammonium. Figure S23. UV-Vis and HRMS of Cu(II) 5,10,15,20-*tetrakis*-(4-carboxyphenyl)porphyrinate, Table S1: Data for Atom Economy calculation, Table S2: Data for E-factor calculation, Table S3: E-factor for the synthesis of Cu(II) complexes of TMePyP, Table S4: E-factor for the synthesis of Cu(II) complexes of 3-OHTPP, Table S5: Data for Ecoscale calculation [1,25,30,31,32,33,34,35,36,37,38,39,40].

## Sample Availability

Samples of the compounds are available from the authors.
