# Supplementary material for: Modern Methods for the Sustainable Synthesis of Metalloporphyrins
Source: Molecules. 2021 Nov 2;26(21):6652. doi: 10.3390/molecules26216652 (PMC8588080; doi:10.3390/molecules26216652)
Supplement: Supplementary file 1 [file molecules-26-06652-s001.zip › molecules-1440322-supplementary.pdf]

Supplementary Materials

Modern methods for the sustainable synthesis of metalloporphyrins

Carla Gomes[1], Mariana Peixoto[1], Marta Pineiro[1,*]

[1] University of Coimbra, Department of Chemistry and CQC, Rua Larga 3004-535, Coimbra, Portugal; carla_sofia.gomes@live.com.pt; mariana.silva.fp@gmail.com; mpineiro@qui.uc.pt

* Correspondence: mpineiro@qui.uc.pt

1. 1. Characterization of the synthesized metalloporphyrins

Zn(II) 5,10,15,20-*tetrakis*-(3,4-dimethoxyphenyl)porphyrinate, Yield = 97%; UV-Vis ($C_4H_8O_2$): λmax, nm (relative absorbance, %) = 426 (100), 551 (17.6), 590 (5.9); ^{1}H-RMN (400 MHz, $CDCl_3$), δ (ppm) = 9.01 (8H, s, H-pyrrole), 7.79 – 7.76 (8H, m, Ph), 7.27 (4H, d, Ph), 4.19 (12H, s, OCH_3), 3.98 (12H, s, OCH_3). Characterization in accordance with previously reported [1].

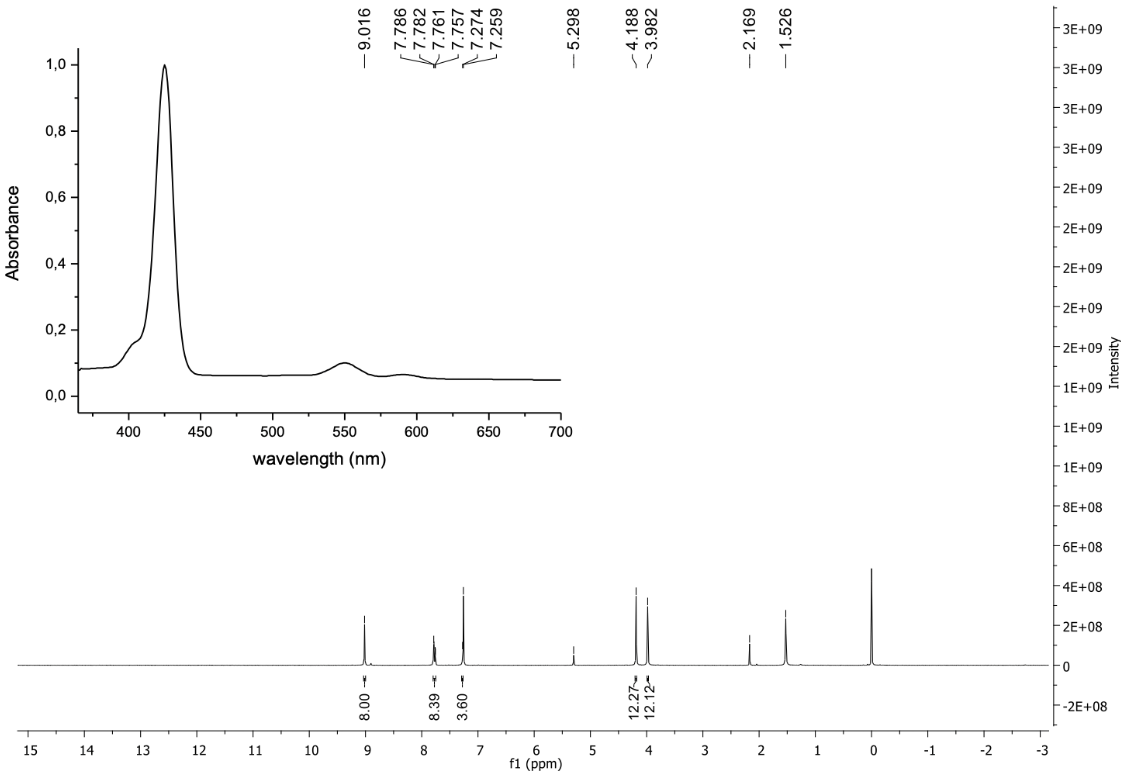

Figure S1. UV-Vis and ^{1}H-NMR spectra of Zn(II) 5,10,15,20-*tetrakis*-(3,4-dimethoxyphenyl)porphyrinate

Cu(II) 5,10,15,20-*tetrakis*-(*N*-methyl-4-pyridinyl)porphyrinate tetraiodide, Yield: 53%; UV-Vis (H_2O): λmax, nm (relative absorbance, %) = 425 (100), 548 (15.6). Characterization in accordance with previously reported [2].

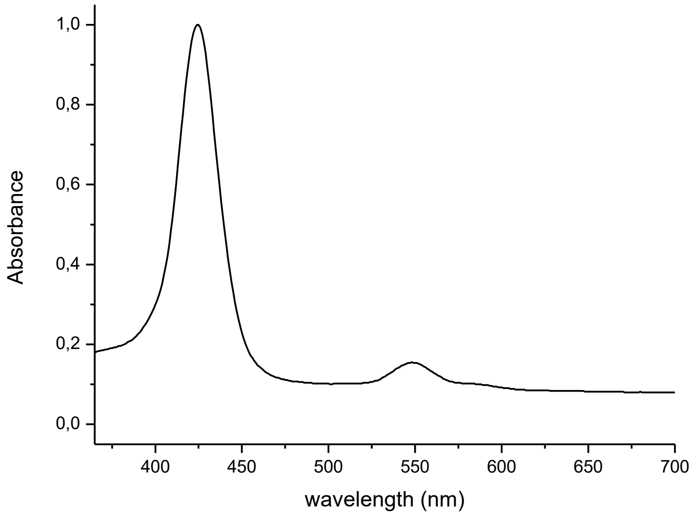

Figure S2. UV-Vis of Cu(II) 5,10,15,20-*tetrakis*-(*N*-methyl-4-pyridinyl)porphyrinate tetraiodide.

Cu(II) 5,10,15,20-*tetrakis*-(3,4-dimethoxyphenyl)porphyrinate, Yield = 84%; UV-Vis ($C_4H_8O_2$): λmax, nm (relative absorbance, %) = 421 (100), 541 (50.8), HRMS (ESI); m/z = 915.2444 ([M+H]$^+$) calculated for 915.2450 ($C_{52}H_{45}CuN_4O_8$). Characterization in accordance with previously reported [1].

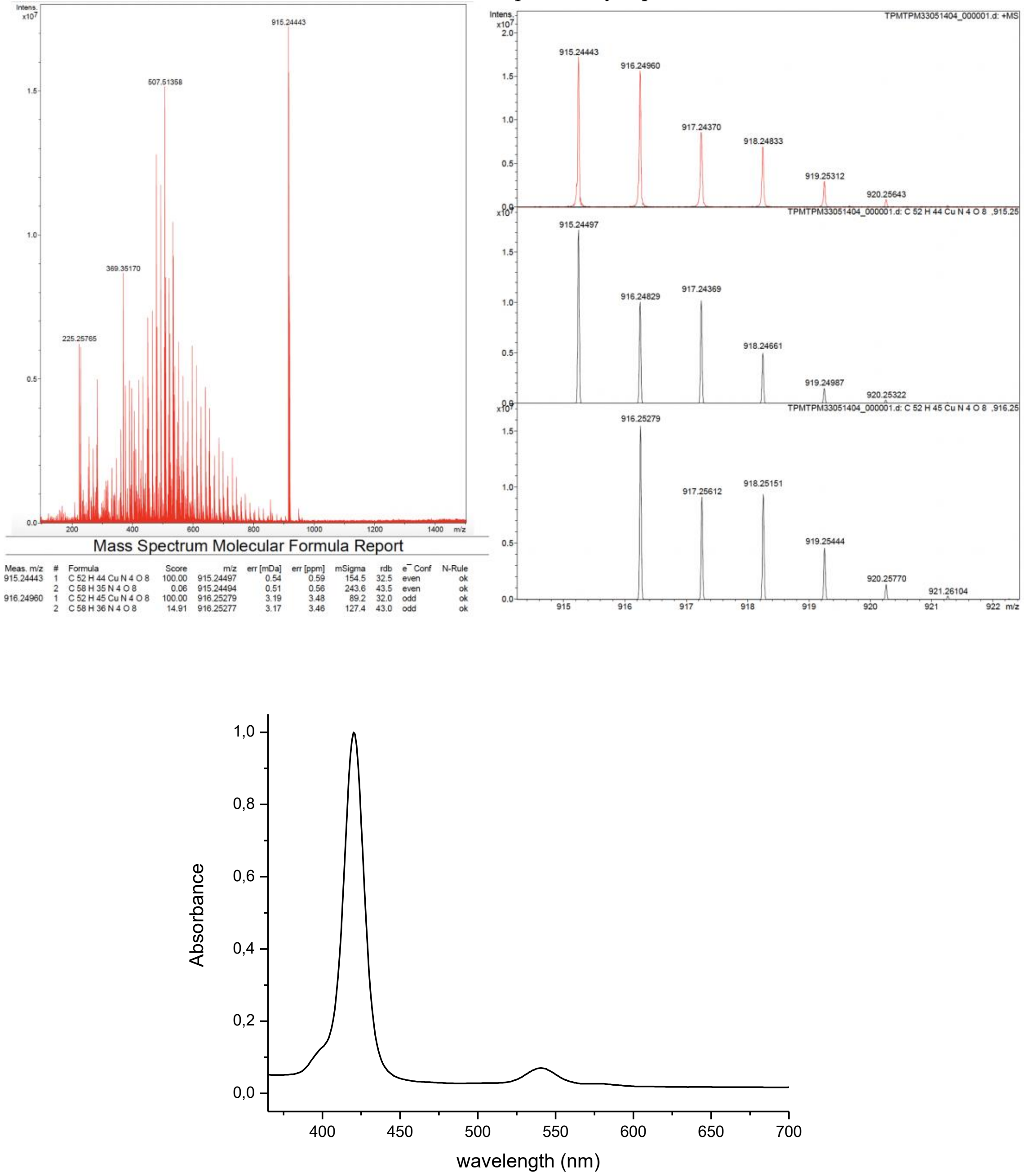

Meas. m/z	#	Formula	Score	m/z	err [mDa]	err [ppm]	mSigma	rdb	e⁻ Conf	N-Rule
915.24443	1	C 52 H 44 Cu N 4 O 8	100.00	915.24497	0.54	0.59	154.5	32.5	even	ok
	2	C 58 H 35 N 4 O 8	0.06	915.24494	0.51	0.56	243.6	43.5	even	ok
916.24960	1	C 52 H 45 Cu N 4 O 8	100.00	916.25279	3.19	3.48	89.2	32.0	odd	ok
	2	C 58 H 36 N 4 O 8	14.91	916.25277	3.17	3.46	127.4	43.0	odd	ok

Figure S3. UV-Vis and HRMS of Cu(II) 5,10,15,20-*tetrakis*-(3,4-dimethoxyphenyl)porphyrinate.

Co(III) 5,10,15,20-*tetrakis*-(3,4-dimethoxyphenyl)porphyrinate acetate, Yield = 61%; UV-Vis (CH_2Cl_2): λmax, nm (relative absorbance, %) = 420 (100), 527 (13.4). Characterization in accordance with previously reported [1].

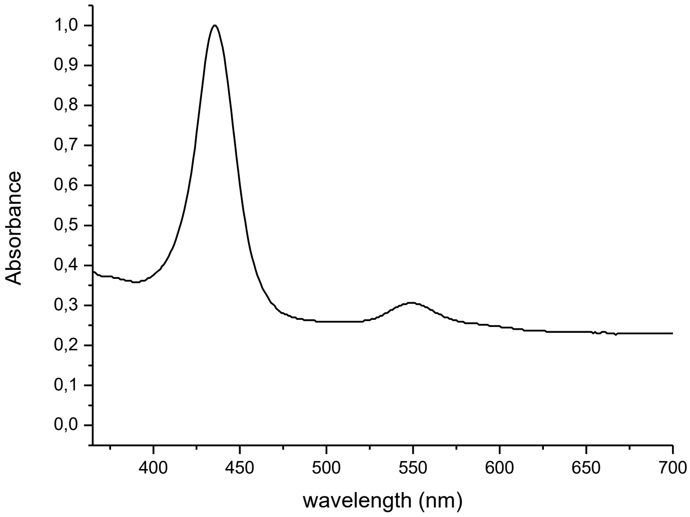

Figure S4. UV-Vis of Co(III) 5,10,15,20-*tetrakis*-(3,4-dimethoxyphenyl)porphyrinate acetate.

Mn(III) 5,10,15,20-(3,4-dimethoxyphenyl)porphyrinate acetate, Yield = 30%; UV-Vis (CH_2Cl_2): λmax, nm (relative absorbance, %) = 482 (100), 586 (14.3), 625 (16.9); HRMS (ESI); m/z = 907.25101 (M+) calculated for 907.25342 ($C_{52}H_{44}MnN_4O_8$).

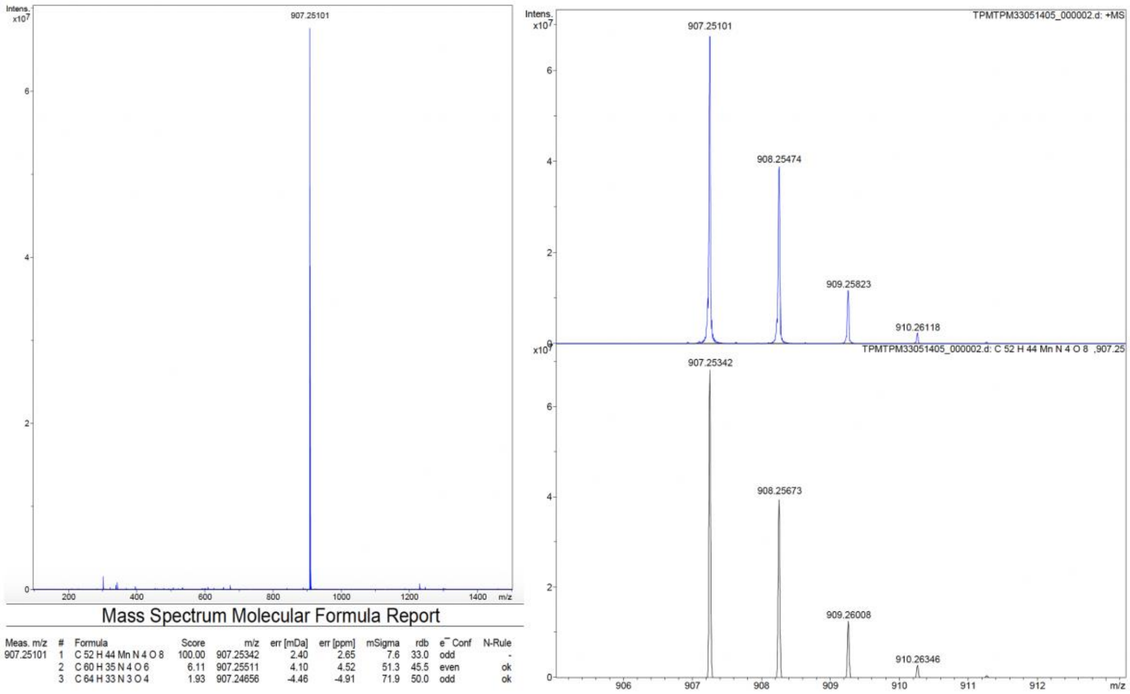

Meas. m/z	#	Formula	Score	m/z	err [mDa]	err [ppm]	mSigma	rdb	e⁻ Conf	N-Rule
907.25101	1	C 52 H 44 Mn N 4 O 8	100.00	907.25342	2.40	2.65	7.6	33.0	odd	-
	2	C 60 H 35 N 4 O 6	6.11	907.25511	4.10	4.52	51.3	45.5	even	ok
	3	C 64 H 33 N 3 O 4	1.93	907.24656	-4.46	-4.91	71.9	50.0	odd	ok

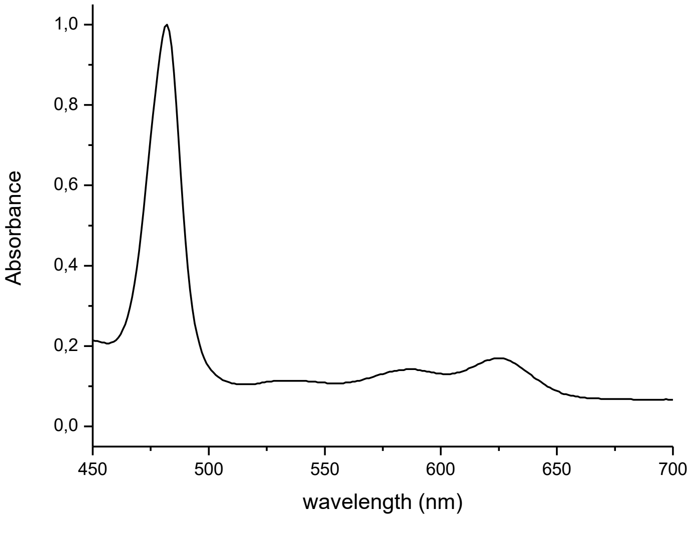

Figure S5. UV-Vis and HRMS spectra of Mn(III) 5,10,15,20-(3,4-dimethoxyphenyl)porphyrinate acetate.

Pd(II) 5,10,15,20-*tetrakis*-(3,4-dimethoxyphenyl)porphyrinate, Yield = 70%; UV-Vis (CH_2Cl_2): λmax, nm (relative absorbance, %) = 422 (100), 525 (9.2); ^{1}H-RMN (400 MHz, $CDCl_3$), δ (ppm) = 8.87 (8H, s, H-pyrrole), 7.73 – 7.71 (8H, m, Ph), 7.25 (4H, d, Ph), 4.17 (12H, s, OCH_3), 3.97 (12H, s, OCH_3).

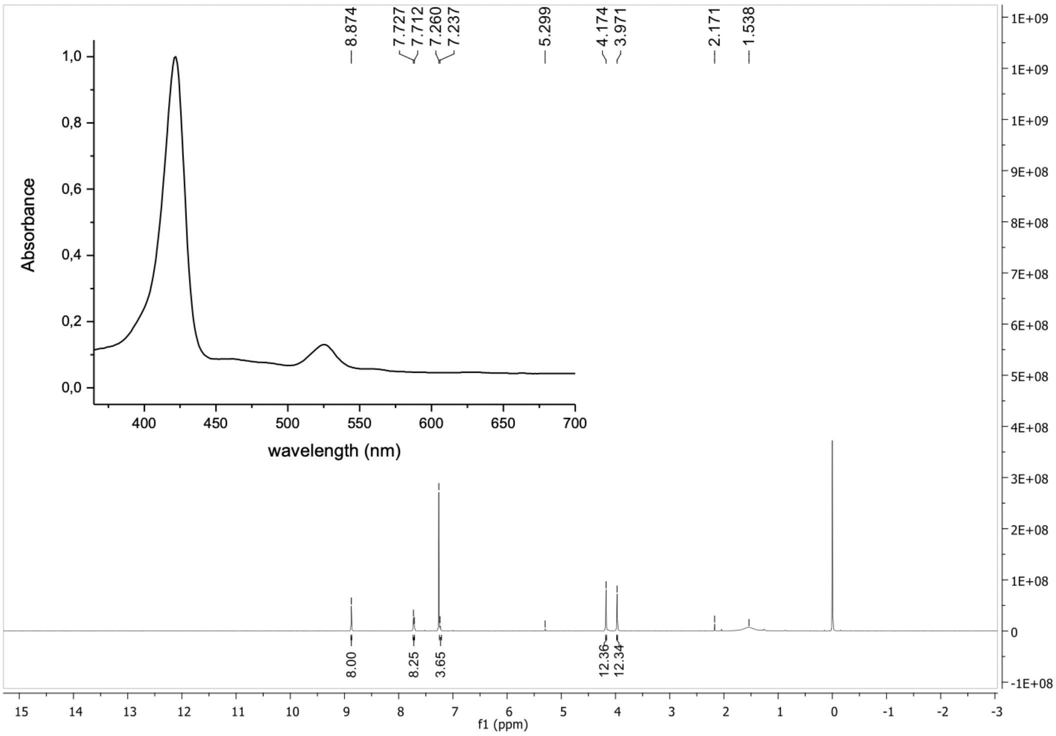

Figure S6. UV-Vis and ^{1}H-NMR spectra of Pd(II) 5,10,15,20-*tetrakis*-(3,4-dimethoxyphenyl)porphyrinate.

Pt(II) 5,10,15,20-(*tetrakis*-3,4-dimethoxyphenyl)porphyrinate, Yield = 50%*; UV-Vis (CH_2Cl_2): λmax, nm (relative absorbance, %) = 424 (100), 541 (50.8); ^{1}H-RMN (400 MHz, $CDCl_3$), δ (ppm) = 8.83 (8H, s, H-pyrrole), 7.63 – 7.61(8H, m, Ph), 7.19 - 7.15 (4H, m, Ph), 4.18 (12H, s, OCH_3), 4.01 (12H, s, OCH_3). *Calculated by NMR.

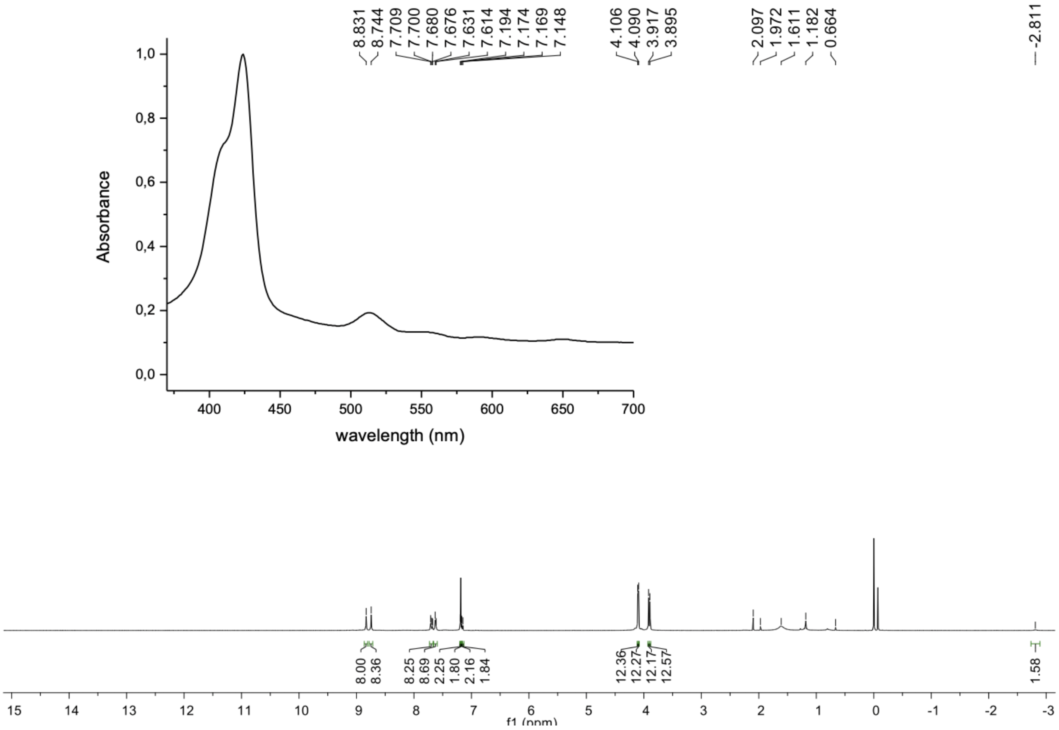

Figure S7. UV-Vis and ^{1}H-NMR spectra of Pt(II) 5,10,15,20-*tetrakis-(*3,4-dimethoxyphenyl)porphyrinate.

Cu(II) 5,10,15,20-tetraphenylporphyrinate, Yield = 75%; UV-Vis ($C_4H_8O_2$): λmax, nm (relative absorbance, %) = 421 (100), 541 (50.8); HRMS (ESI); m/z = 675.1593 (M^+) calculated for 675.1605 ($C_{44}H_{28}CuN_4$). Characterization in accordance with previously reported [3, 4].

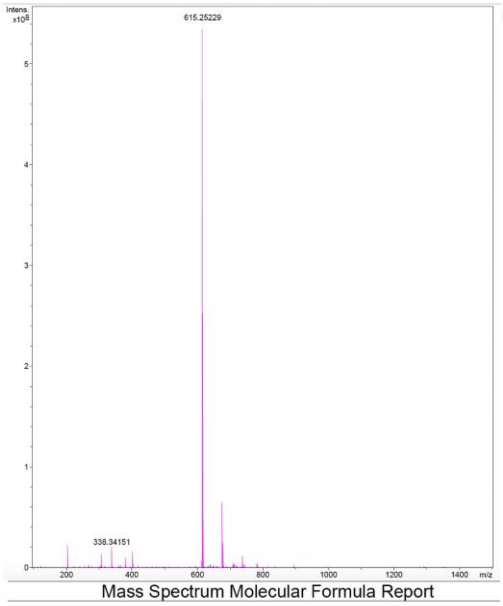

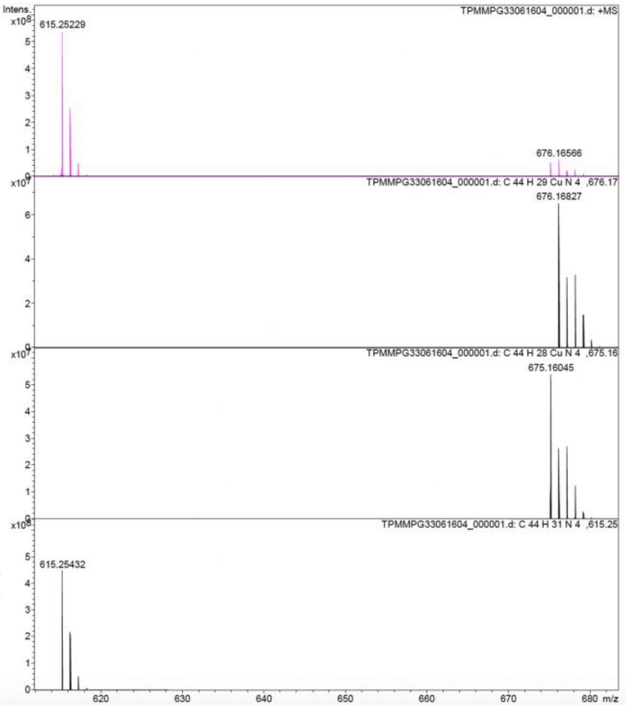

Mass Spectrum Molecular Formula Report

Meas. m/z	#	Formula	Score	m/z	err [mDa]	err [ppm]	mSigma	rdb	e⁻ Conf	N-Rule
615.25229	1	C 44 H 31 N 4	100.00	615.25432	2.04	3.31	21.2	31.5	even	ok
	2	C 38 H 40 Cu N 4	0.00	615.25435	2.06	3.35	235.2	20.5	even	ok
675.15929	1	C 44 H 28 Cu N 4	100.00	675.16045	1.16	1.72	249.9	32.5	even	ok
	2	C 50 H 19 N 4	0.05	675.16042	1.13	1.68	315.1	43.5	even	ok
676.16566	1	C 50 H 20 N 4	100.00	676.16825	2.59	3.83	129.3	43.0	odd	ok
	2	C 44 H 29 Cu N 4	67.19	676.16827	2.62	3.87	135.6	32.0	odd	ok

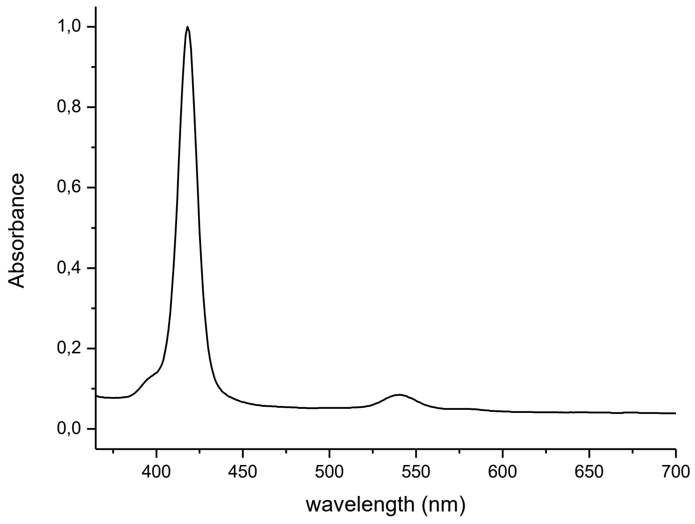

Figure S8. UV-Vis and HRMS of Cu(II) 5,10,15,20-tetraphenylporphyrinate.

Cu(II) 5,10,15,20-*tetrakis*-(3,5-dimethoxyphenyl)porphyrinate, Yield = 89%; UV-Vis ($C_4H_8O_2$): λmax, nm (relative absorbance, %) = 417 (100), 538 (4.3); HRMS (ESI); m/z = 916.2508 ($[M+H]^+$) calculated for 916.2530 ($C_{52}H_{45}CuN_4O_8$). Characterization in accordance with previously reported [5].

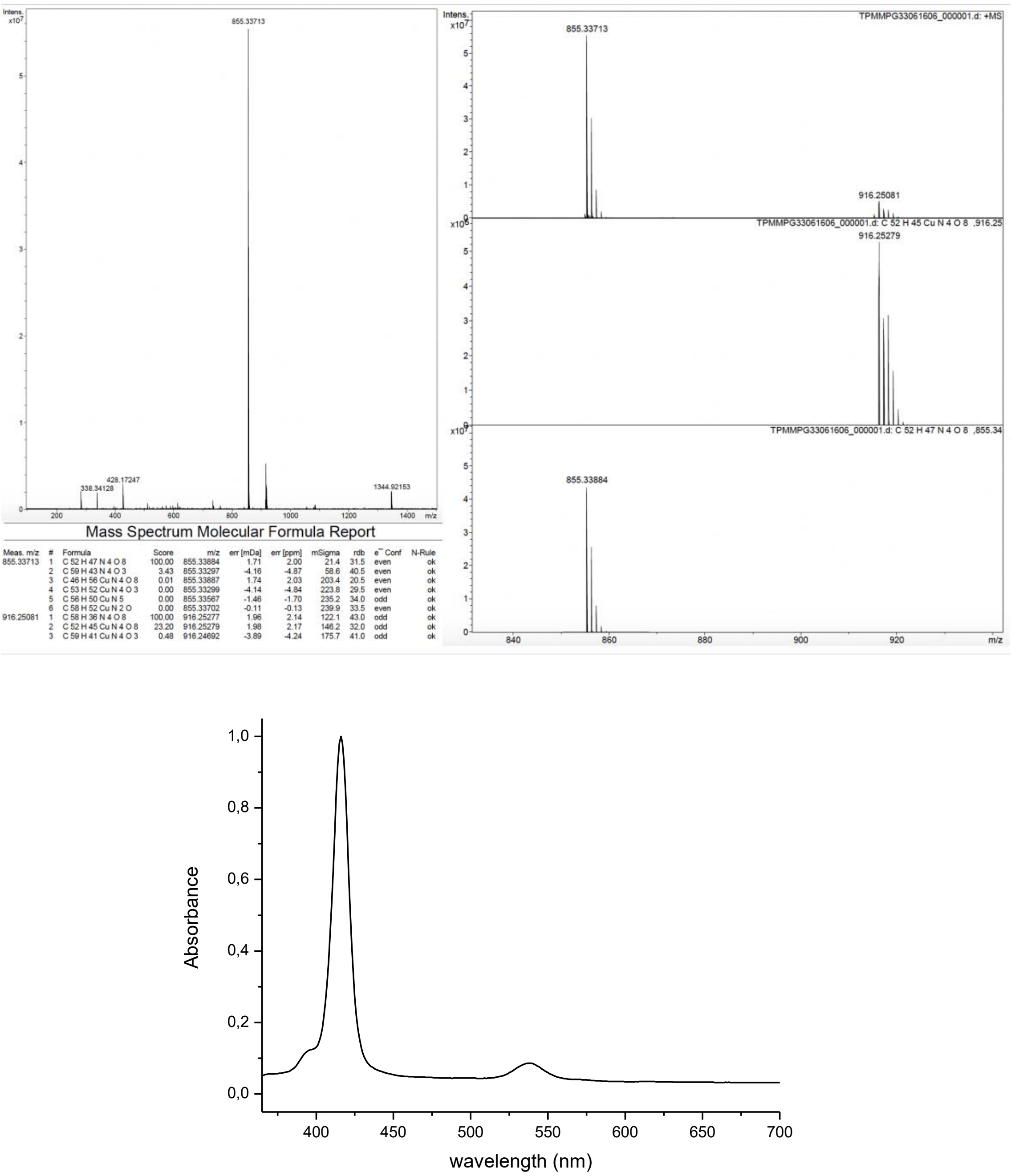

Figure S9. UV-Vis and HRMS of Cu(II) 5,10,15,20-*tetrakis*-(3,5-dimethoxyphenyl)porphyrinate.

Cu(II) 5,10,15,20-*tetrakis*-(3,4,5-trimethoxyphenyl)porphyrinate, Yield = 88%; UV-Vis (C_2H_3N): λmax, nm (relative absorbance, %) = 416 (100), 536 (5); HRMS (ESI); m/z = 1035.2850 (M^+) calculated for 1035.2872 ($C_{56}H_{52}CuN_4O_{12}$). Characterization in accordance with previously reported [6].

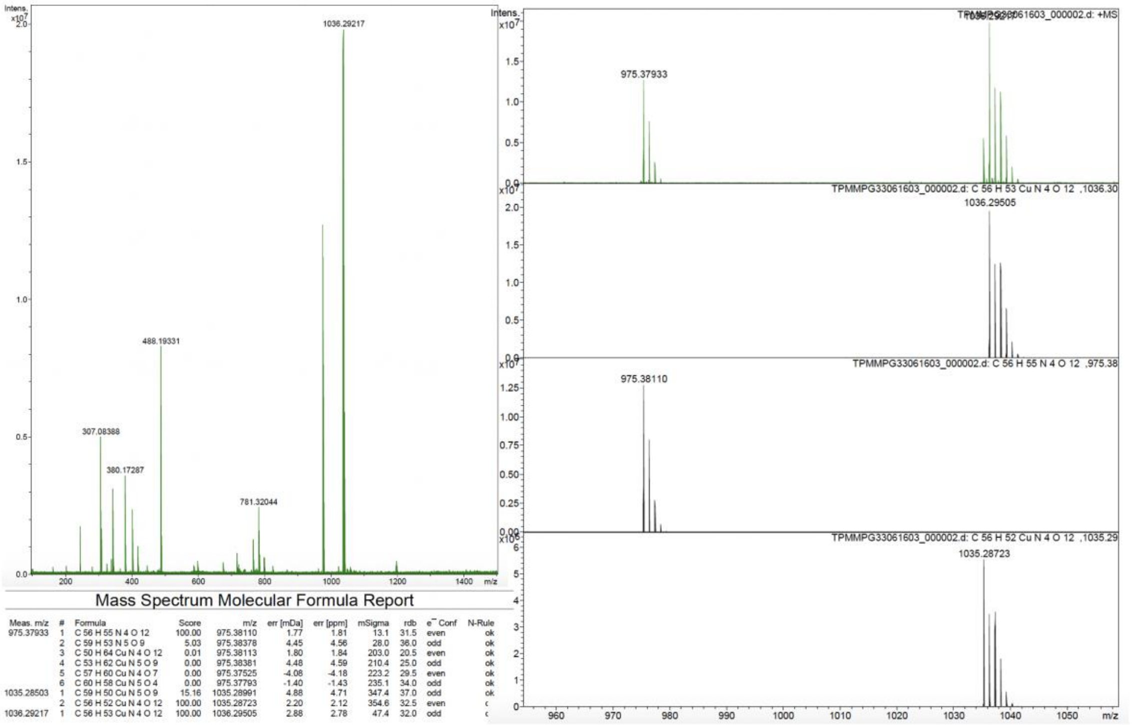

Meas. m/z	#	Formula	Score	m/z	err [mDa]	err [ppm]	mSigma	rdb	e⁻ Conf	N-Rule
975.37933	1	C 56 H 55 N 4 O 12	100.00	975.38110	1.77	1.81	13.1	31.5	even	ok
	2	C 59 H 53 N 5 O 9	5.03	975.38378	4.45	4.56	28.0	36.0	odd	ok
	3	C 50 H 64 Cu N 4 O 12	0.01	975.38113	1.80	1.84	203.0	20.5	even	ok
	4	C 53 H 62 Cu N 5 O 9	0.00	975.38381	4.48	4.59	210.4	25.0	odd	ok
	5	C 57 H 60 Cu N 4 O 7	0.00	975.37525	-4.08	-4.18	223.2	29.5	even	ok
	6	C 60 H 58 Cu N 5 O 4	0.00	975.37793	-1.40	-1.43	235.1	34.0	odd	ok
1035.28503	1	C 59 H 50 Cu N 5 O 9	15.16	1035.28991	4.88	4.71	347.4	37.0	odd	ok
	2	C 56 H 52 Cu N 4 O 12	100.00	1035.28723	2.20	2.12	354.6	32.5	even	[illegible]
1036.29217	1	C 56 H 53 Cu N 4 O 12	100.00	1036.29505	2.88	2.78	47.4	32.0	odd	[illegible]

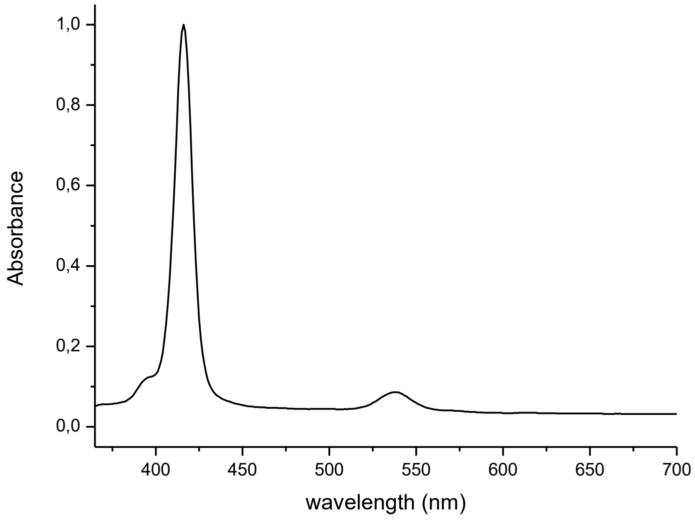

Figure S10. UV-Vis and HRMS of Cu(II) 5,10,15,20-*tetrakis*-(3,4,5-trimethoxyphenyl)porphyrinate.

Cu(II) 5,10,15,20-*tetrakis*-(3-hydroxyphenyl)porphyrinate, Yield = 74%; UV-Vis (CH_3OH): λmax, nm (relative absorbance, %) = 414 (100), 537 (6.2). Characterization in accordance with previously reported [6, 7].

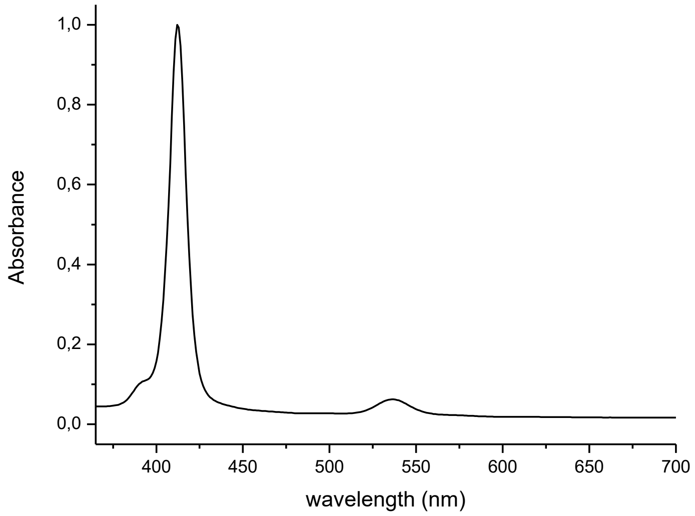

Figure S11. UV-Vis of Cu(II) 5,10,15,20-*tetrakis*-(3-hydroxyphenyl)porphyrinate.

Cu(II) 5,10,15,20-*tetrakis*-(3-hydroxyphenyl-4-methoxyphenyl))porphyrinate, Yield = 90%; UV-Vis ($C_4H_8O_2$): λmax, nm (relative absorbance, %) = 405 (100), 481 (34.4), 530 (5.4); HRMS (ESI); m/z = 859.1817 (M^+) $^+$) calculated for 859.1824 ($C_{48}H_{36}CuN_4O_8$). Characterization in accordance with previously reported [8].

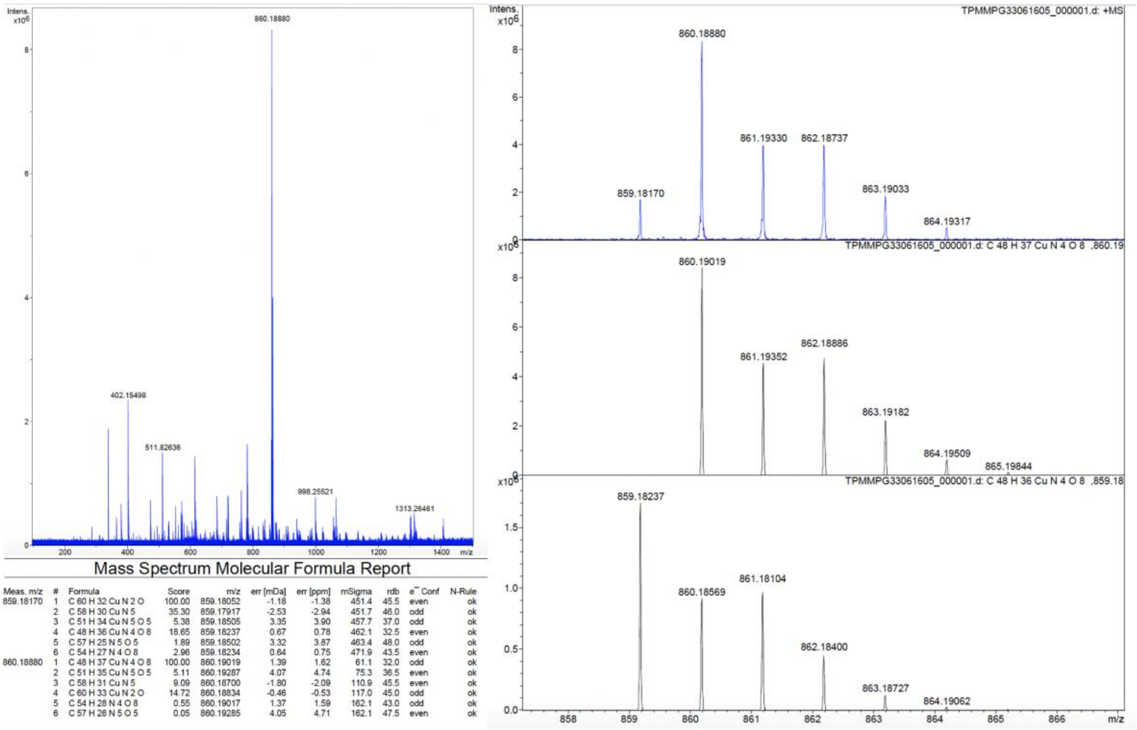

Mass Spectrum Molecular Formula Report

Meas. m/z	#	Formula	Score	m/z	err [mDa]	err [ppm]	mSigma	rdb	e⁻ Conf	N-Rule
859.18170	1	C 60 H 32 Cu N 2 O	100.00	859.18052	-1.18	-1.38	451.4	45.5	even	ok
	2	C 58 H 30 Cu N 5	35.30	859.17917	-2.53	-2.94	451.7	46.0	odd	ok
	3	C 51 H 34 Cu N 5 O 5	5.38	859.18505	3.35	3.90	457.7	37.0	odd	ok
	4	C 48 H 36 Cu N 4 O 8	18.65	859.18237	0.67	0.78	462.1	32.5	even	ok
	5	C 57 H 25 N 5 O 5	1.89	859.18502	3.32	3.87	463.4	48.0	odd	ok
	6	C 54 H 27 N 4 O 8	2.96	859.18234	0.64	0.75	471.9	43.5	even	ok
860.18880	1	C 48 H 37 Cu N 4 O 8	100.00	860.19019	1.39	1.62	61.1	32.0	odd	ok
	2	C 51 H 35 Cu N 5 O 5	5.11	860.19287	4.07	4.74	75.3	36.5	even	ok
	3	C 58 H 31 Cu N 5	9.09	860.18700	-1.80	-2.09	110.9	45.5	even	ok
	4	C 60 H 33 Cu N 2 O	14.72	860.18834	-0.46	-0.53	117.0	45.0	odd	ok
	5	C 54 H 28 N 4 O 8	0.55	860.19017	1.37	1.59	162.1	43.0	odd	ok
	6	C 57 H 26 N 5 O 5	0.05	860.19285	4.05	4.71	162.1	47.5	even	ok

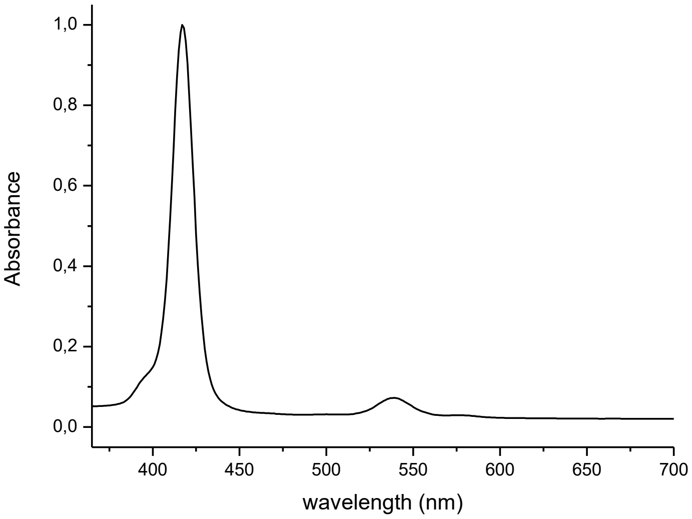

Figure S12. UV-Vis and HRMS of Cu(II) 5,10,15,20-*tetrakis*-(3-hydroxyphenyl-4-methoxyphenyl))porphyrinate.

Cu(II) 5,10,15,20-*tetrakis*-(3,5-dichlorophenyl)porphyrinate, Yield = 97%; UV-Vis ($C_4H_8O_2$): λmax, nm (relative absorbance, %) = 415 (100), 539 (5.6), 587 (2.2); HRMS (ESI); m/z = 947.8553 ($[M+H]^+$) calculated for 947.8565 ($C_{44}H_{21}Cl_8CuN_4$).

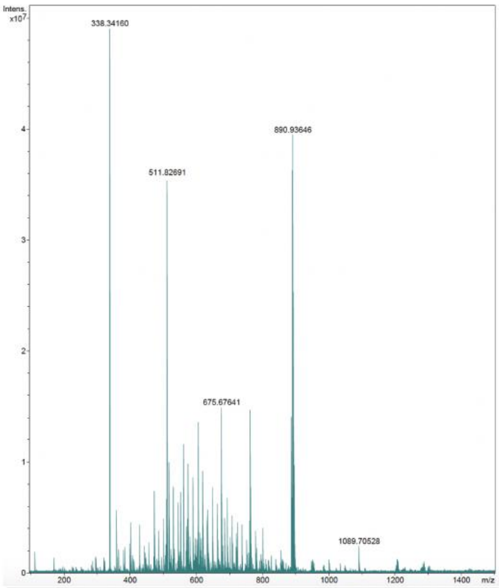

Mass Spectrum Molecular Formula Report

Meas. m/z	#	Formula	Score	m/z	err [mDa]	err [ppm]	mSigma	rdb	e⁻ Conf	N-Rule
886.94295	1	C 44 H 23 Cl 8 N 4	100.00	886.94254	-0.41	-0.46	35.2	31.5	even	ok
	2	C 50 H 21 Cl 5 Cu N 2	14.58	886.94379	0.84	0.95	85.5	38.5	even	ok
	3	C 38 H 32 Cl 8 Cu N 4	8.06	886.94257	-0.38	-0.43	103.5	20.5	even	ok
	4	C 51 H 16 Cl 4 Cu N 4	0.13	886.94196	-0.99	-1.12	166.4	43.5	even	ok
	5	C 56 H 12 Cl 5 N 2	0.04	886.94376	0.81	0.92	183.2	49.5	even	ok
947.85527	1	C 50 H 12 Cl 8 N 4	100.00	947.85647	1.20	1.26	63.1	43.0	odd	ok
	2	C 44 H 21 Cl 8 Cu N 4	43.25	947.85650	1.22	1.29	84.5	32.0	odd	ok
	3	C 56 H 10 Cl 5 Cu N 2	0.79	947.85771	2.44	2.58	143.8	50.0	odd	ok

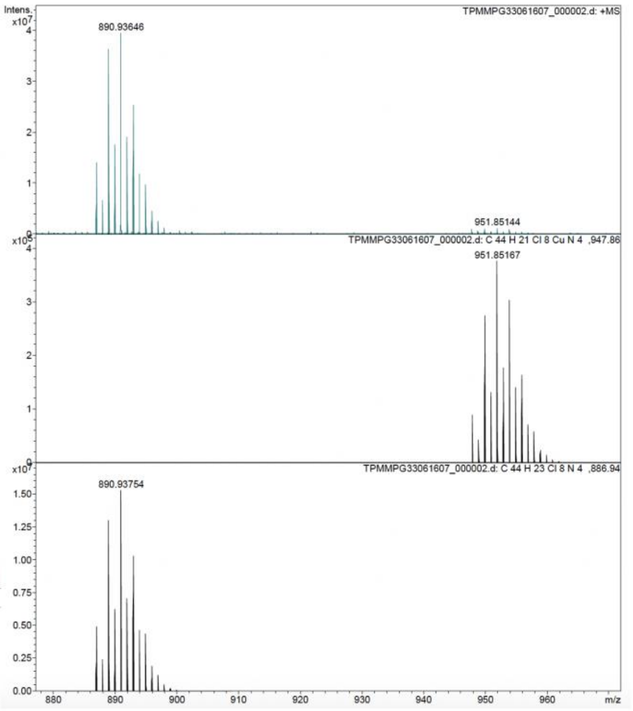

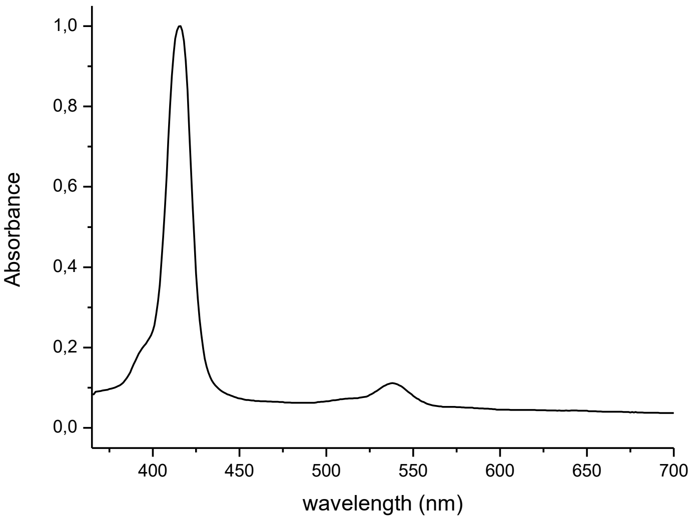

Figure S13. UV-Vis and HRMS of Cu(II) 5,10,15,20-*tetrakis*-(3,5-dichlorophenyl)porphyrinate.

Cu(II) 5,10,15,20-*tetrakis*-(3-nitrophenyl)porphyrinate, Yield = 70%; UV-Vis (CH_2Cl_2): λmax, nm (relative absorbance, %) = 418 (100), 539 (14.9). Characterization in accordance with previously reported [9].

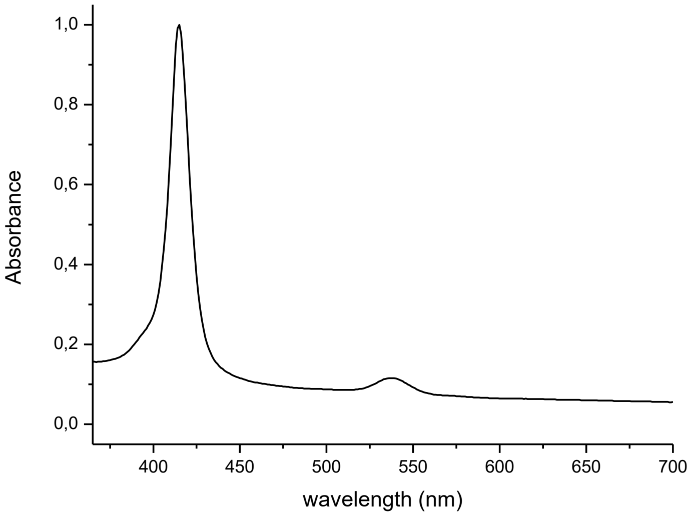

Figure S14. UV-Vis and of Cu(II) 5,10,15,20-*tetrakis*-(3-nitrophenyl)porphyrinate.

Zn(II) 5,10,15,20-*tetrakis*-(*N*-methyl-4-pyridinyl)porphyrinate tetraiodide, Yield = 90%; UV-Vis (H_2O): λmax, nm (relative absorbance, %) = 436 (100), 564 (16.1), 604 (11.3); ^{1}H-RMN (400 MHz, DMSO-d_6), δ (ppm) = 9.43 (8H, d, J = 6.8 Hz, Ph), 9.09 (8H, s, H-pyrrole), 8.92 (8H, d, J = 6.4 Hz, CH), 4.73 (12H, s, CH_3). Characterization in accordance with previously reported [10].

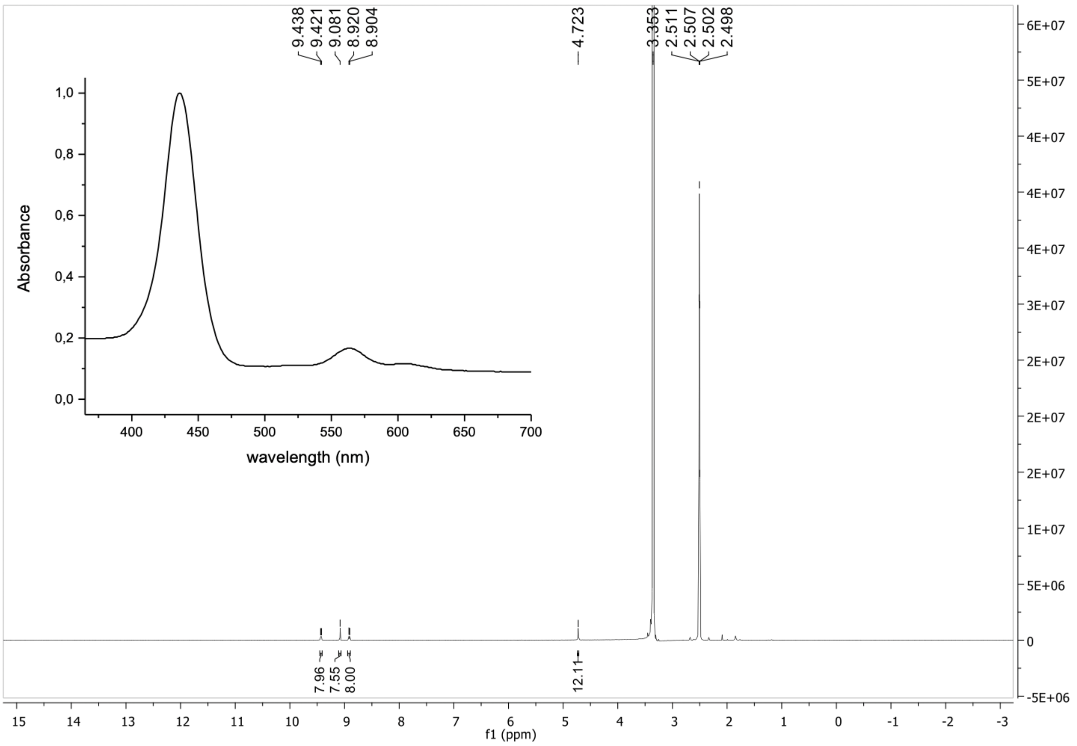

Figure S15. UV-Vis and ^{1}H-NMR of Zn(II) 5,10,15,20-*tetrakis*-(*N*-methyl-4-pyridinyl)porphyrinate tetraiodide.

Mn(III) acetate 5,10,15,20-*tetrakis*-(*N*-methyl-4-pyridinyl)porphyrinate tetraiodide, Yield = 18%; UV-Vis (H_2O): λmax, nm (relative absorbance, %) = 462 (100), 558 (29.0). Characterization in accordance with previously reported [11].

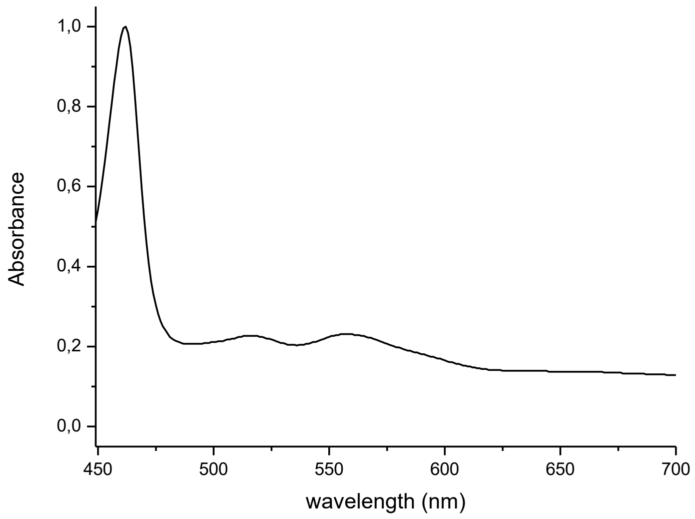

Figure S16. UV-Vis of Mn(III) 5,10,15,20-*tetrakis*-(*N*-methyl-4-pyridinyl)porphyrinate tetraiodide.

Ni(II) 5,10,15,20-*tetrakis*-(*N*-methyl-4-pyridinyl)porphyrinate tetraiodide, Yield = 18%; UV-Vis (H_2O): λmax, nm (relative absorbance, %) = 423 (100), 522 (25.1), 558 (24.0). Characterization in accordance with previously reported [12].

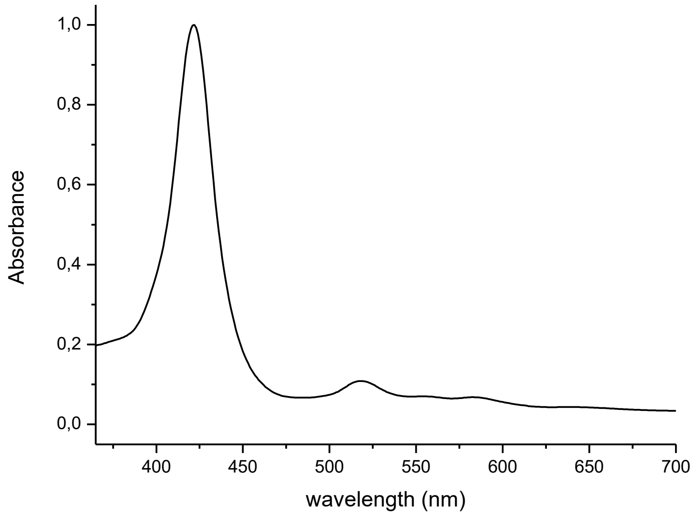

Figure S17. UV-Vis of Ni(II) 5,10,15,20-*tetrakis*-(*N*-methyl-4-pyridinyl)porphyrinate tetraiodide.

Co(III) 5,10,15,20-*tetrakis*-(*N*-methyl-4-pyridinyl)porphyrinate tetraiodide, Yield = 32%; UV-Vis (H_2O): λmax, nm (relative absorbance, %) = 435 (100), 548 (30.7). Characterization in accordance with previously reported [12].

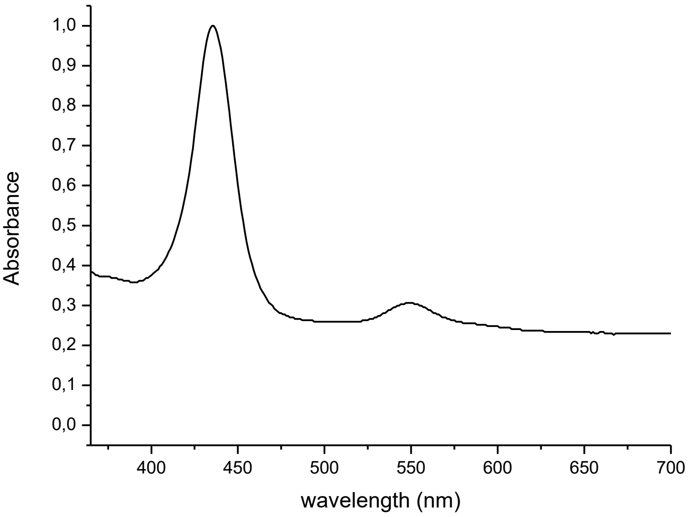

Figure S18. UV-Vis of Co(III) 5,10,15,20-*tetrakis*-(*N*-methyl-4-pyridinyl)porphyrinate tetraiodide.

Zn(II) 5,10,15,20-*tetrakis*-(4-phosphonatophenyl)porphyrinate, Yield = 32%; UV-Vis (H_2O): λmax, nm (relative absorbance, %) = 413 (100), 539 (8.5); ^{1}H-RMN (400 MHz, DMSO-d_6), δ (ppm) = 9.03 - 9.00 (8H, m, H-pyrrole), 7.75 - 7.73 (8H, m, Ph), 7.69 - 7.66 (2H, m, Ph).

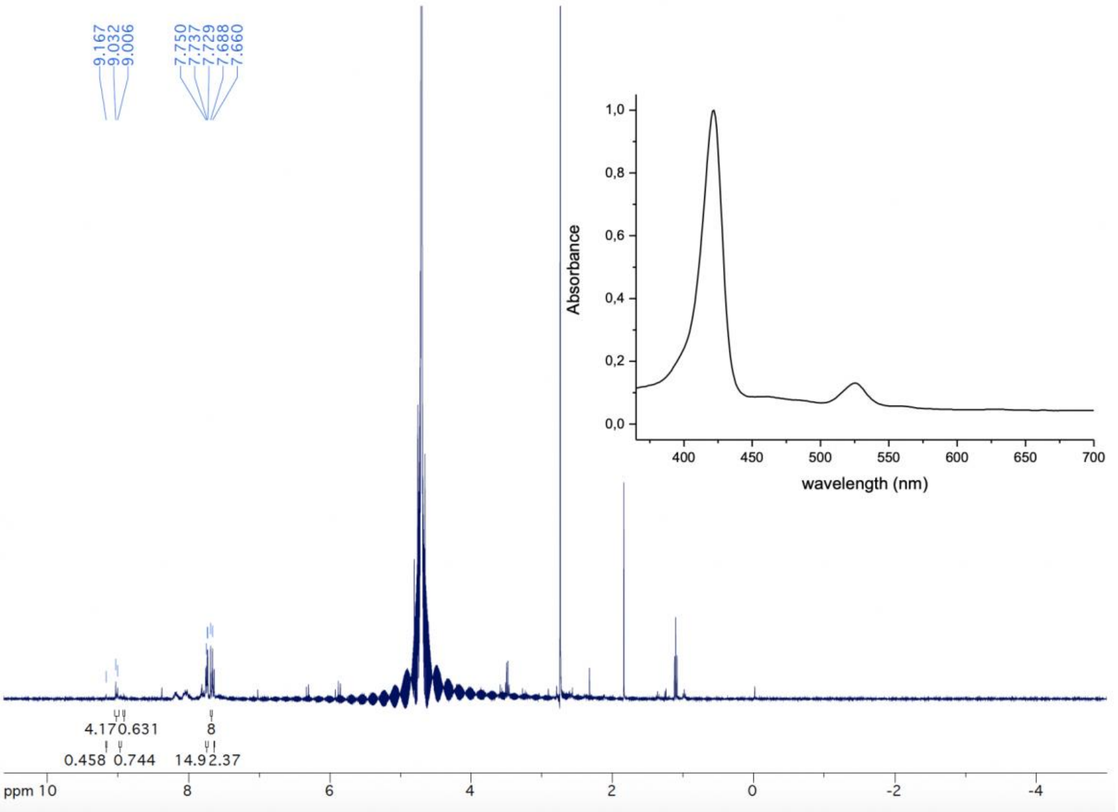

Figure S19. UV-Vis and ^{1}H-NMR of Zn(II) 5,10,15,20-*tetrakis*-(4-phosphonatophenyl)porphyrinate.

Zn(II) 5,10,15,20-*tetrakis*-(4-carboxyphenyl)porphyrinate, Yield = 46%; UV-Vis (CH_3OH): λmax, nm (relative absorbance, %) = 422 (100), 556 (6), 596 (4.2); ^{1}H-RMN (400 MHz, D_2O), δ (ppm) = 8.98 (8H, s, H-pyrrole), 8.25 (8H, d, J = 7.2 Hz, Ph), 7.91 (8H, d, J = 6.8 Hz, Ph), 7.80 (4H, s, CO_2H) Characterization in accordance with previously reported [13].

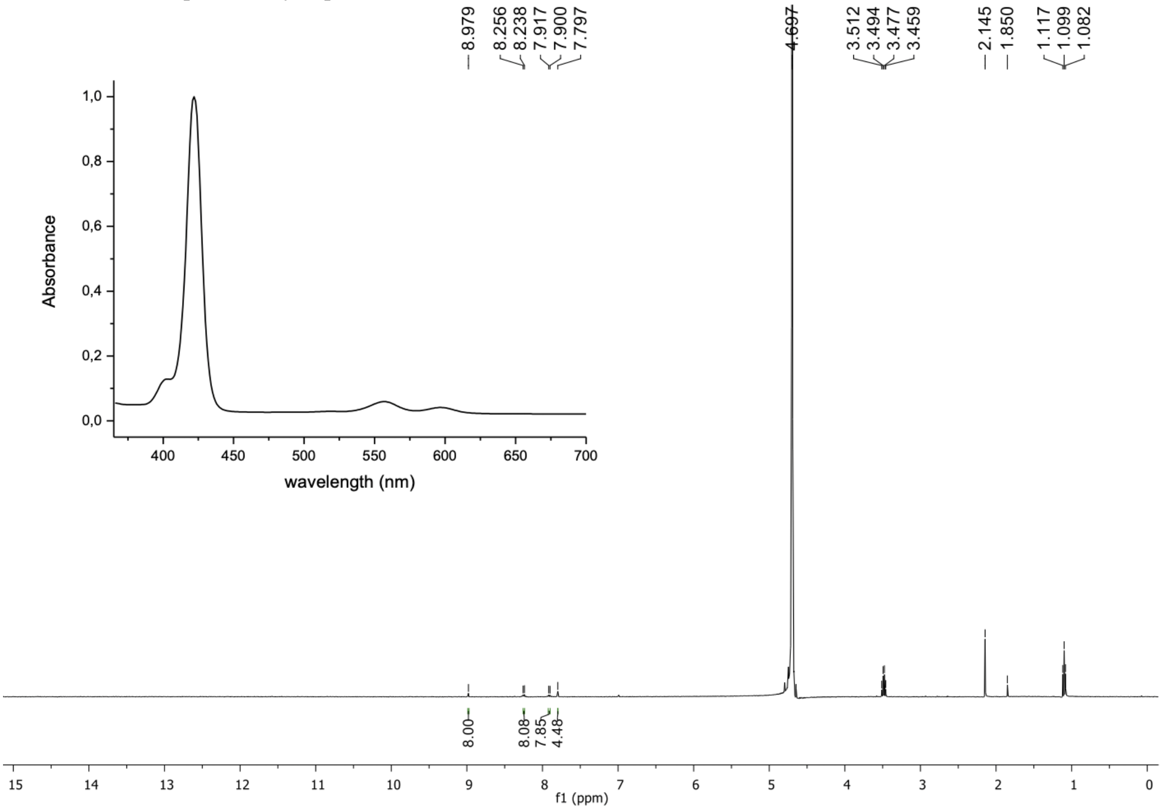

Figure S20. UV-Vis and ^{1}H-NMR of Zn(II) 5,10,15,20-*tetrakis*-(4-carboxyphenyl)porphyrinate.

Zn(II) 5,10,15,20-*tetrakis*-(3-hydroxyphenyl)porphyrinate, Yield = 89%; UV-Vis (CH_3OH): λmax, nm (relative absorbance, %) = 421 (100), 553 (19.3); ^{1}H-RMN (400 MHz, DMSO-d_6), δ (ppm) = 9.79 (4H, s, OH), 8.83 (8H, s, H-pyrrole), 7.62 – 7.54 (12H, m, Ph), 7.21 - 7.19(4H, m, Ph).

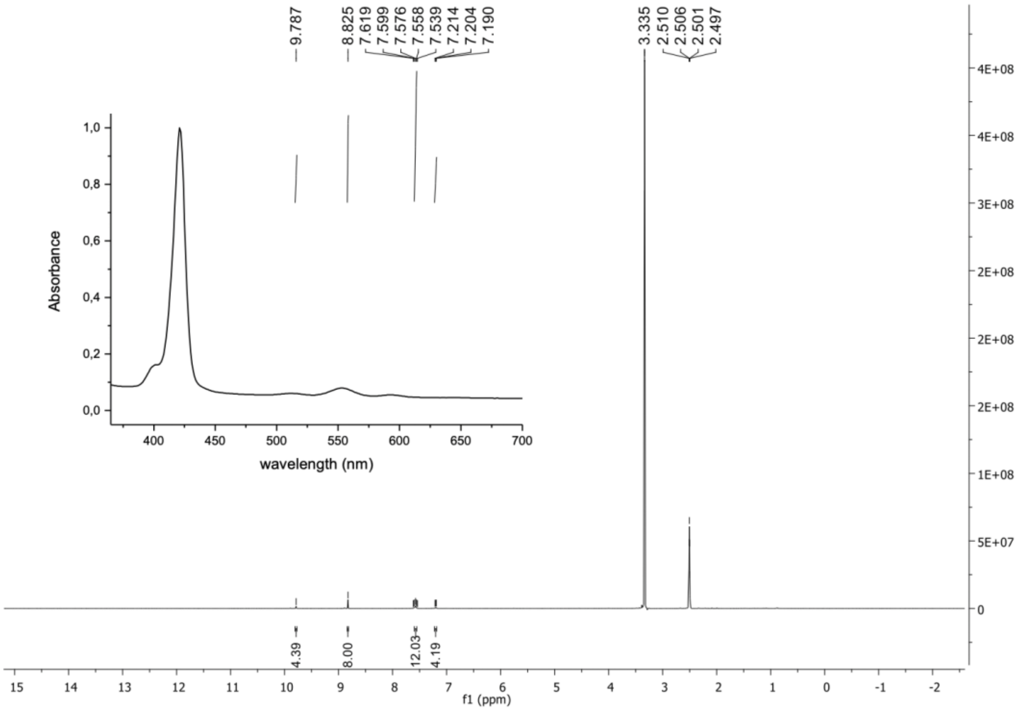

Figure S21. UV-Vis and ^{1}H-NMR of Zn(II) 5,10,15,20-*tetrakis*-(3-hydroxyphenyl)porphyrinate.

Zn(II) 5,10,15,20- *tetrakis*-(4-sulfonatophenyl)porphyrinato tetraammonium, Yield = 65%; UV-Vis (CH_3OH): λmax, nm (relative absorbance, %) = 420 (100), 556 (14.5) 596 (13); ^{1}H-RMN (400 MHz, DMSO-d_6), δ (ppm) = 8.80-8.76 (8H, m, H-pyrrole), 8.20-8.18 (4H, m, Ph), 8.13 (4H, d, J = 8.0 Hz, Ph), 8.02 (4H, d, J = 8.4 Hz, Ph), 7.81-7.79 (4H, m, Ph) (Characterization in accordance with previously reported [13].

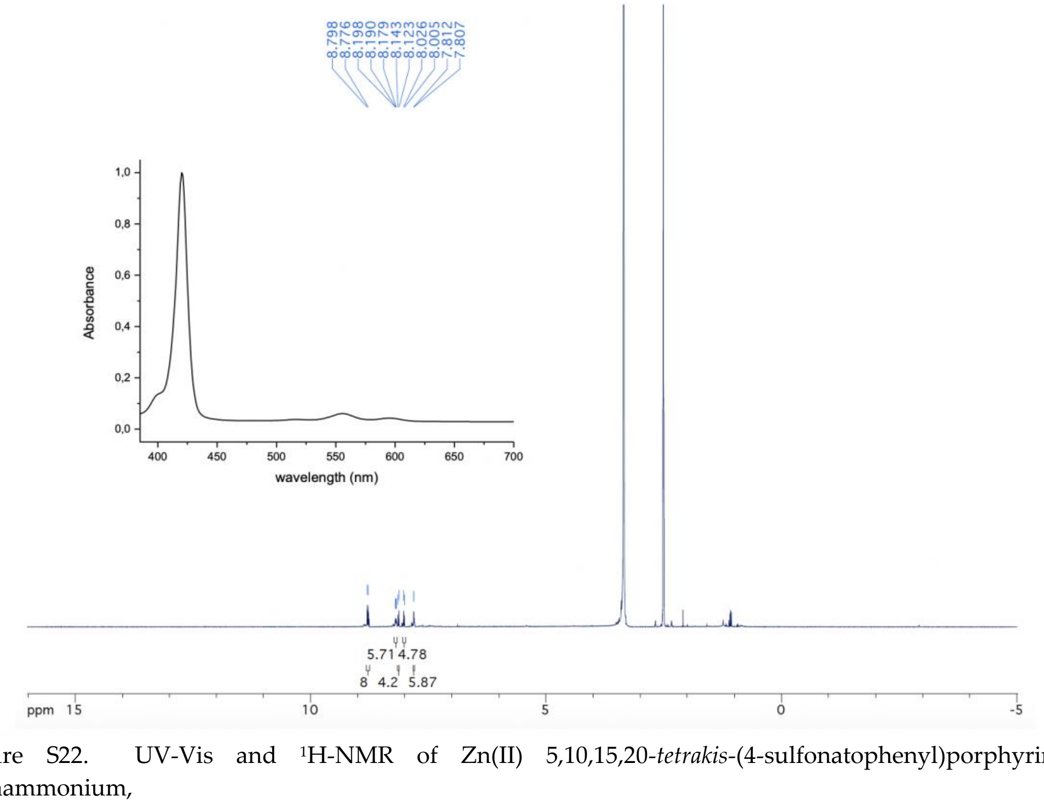

Figure S22. UV-Vis and ^{1}H-NMR of Zn(II) 5,10,15,20-*tetrakis*-(4-sulfonatophenyl)porphyrinato tetraammonium,

Cu(II) 5,10,15,20-*tetrakis*-(4-carboxyphenyl)porphyrinate, Yield = 46%; UV-Vis (CH_3OH): λmax, nm (relative absorbance, %) = 413 (100), 539 (10.6); HRMS (ESI); m/z = 852.1298 (M+) calculated for 852.1276 ($C_{48}H_{29}CuN_4O_8$). Characterization in accordance with previously reported[10].

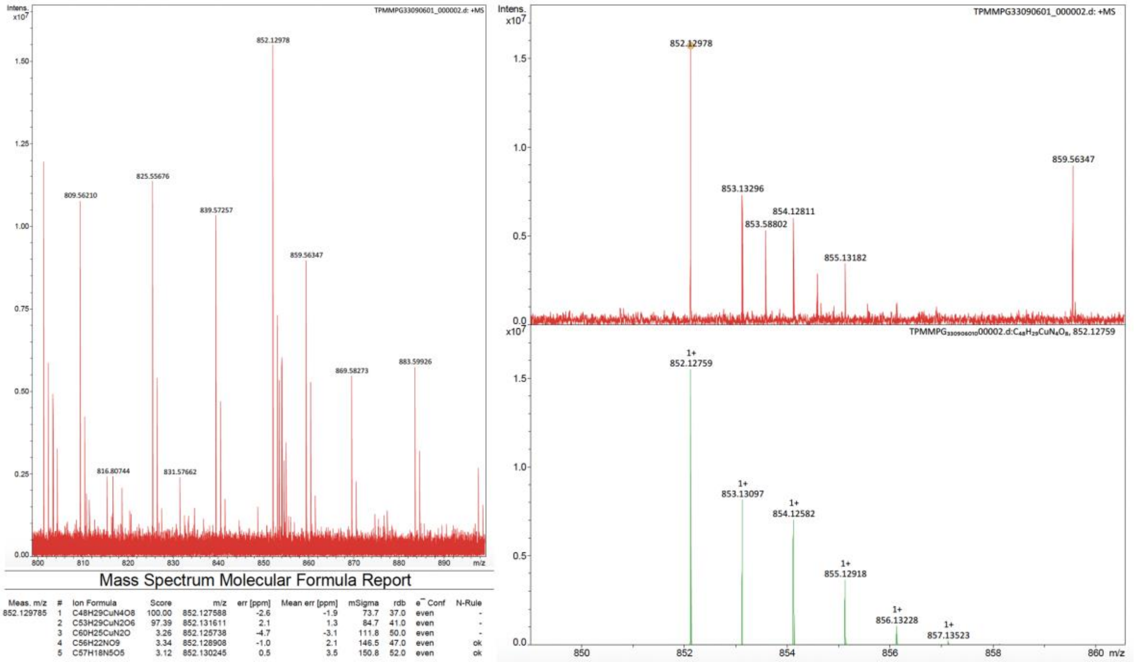

Meas. m/z	#	Ion Formula	Score	m/z	err [ppm]	Mean err [ppm]	mSigma	rdb	e⁻ Conf	N-Rule
852.129785	1	C48H29CuN4O8	100.00	852.127588	-2.6	-1.9	73.7	37.0	even	-
	2	C53H29CuN2O6	97.39	852.131611	2.1	1.3	84.7	41.0	even	-
	3	C60H25CuN2O	3.26	852.125738	-4.7	-3.1	111.8	50.0	even	-
	4	C56H22NO9	3.34	852.128908	-1.0	2.1	146.5	47.0	even	ok
	5	C57H18N5O5	3.12	852.130245	0.5	3.5	150.8	52.0	even	ok

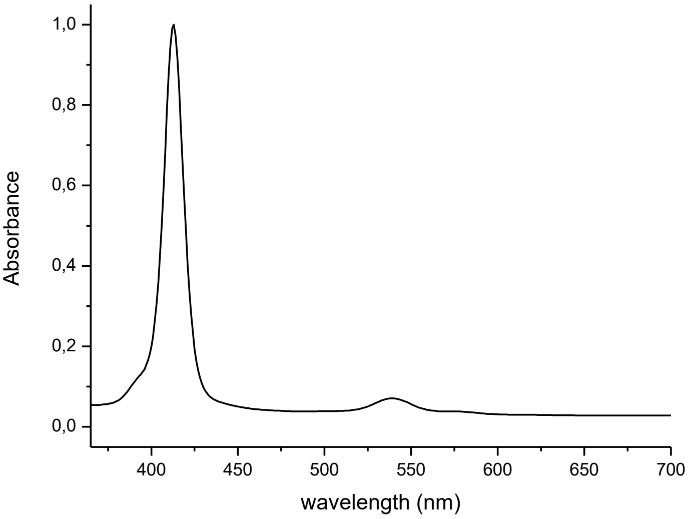

Figure S23. UV-Vis and HRMS of Cu(II) 5,10,15,20-*tetrakis*-(4-carboxyphenyl)porphyrinate.

2. Sustainability assessment

2.1. Experimental procedures of the reactions used for the sustainability calculations

2.1.1. Synthesis of copper (II) 5,10,15,20-*tetrakis*-(*N*-methyl-4-pyridinyl)porphyrinate, CuTMePyP under mechanical action

A mixture of 5,10,15,20-*tetrakis*-(*N*-methyl-4-pyridinyl)porphyrin (50 mg, 0.042 mmol) and copper(II) acetate monohydrate (10 equiv, 84.2 mg) and two steel spheres were placed in steel jar and submitted to mechanical

action in a ball milling system (Retsch 400 MM) at 25 Hz during 60 min. The reaction product was purified through exclusion chromatography (Sephadex G-10) using water as eluent. Yield 45%.

2.1.2 Synthesis of copper (II) 5,10,15,20-*tetrakis*-(3-hydroxyphenyl)porphyrinate under ultrasound
A mixture of 5,10,15,20-*tetrakis*-(3-hydroxyphenyl)porphyrin (50 mg, 0.074 mmol), copper(II) acetate monohydrate (5 equiv, 80.2 mg) and two steel spheres were placed in steel jar and submitted to mechanical action in a ball milling system (Retsch 400 MM) at 25 Hz during 30 min. After liquid-liquid extraction (ethyl acetate/water) and solvent evaporation the Cu(II) 5,10,15,20-*tetrakis*-(3-hydroxyphenyl)porphyrinate was obtained in 74% yield.

2.1.3. Synthesis of copper(II) 5,10,15,20-*tetrakis*-(*N*-methyl-4-pyridinyl)porphyrinate, CuTMePyP under ultrasound
A mixture of 5,10,15,20-*tetrakis*-(*N*-methyl-4-pyridinyl)porphyrin (50 mg, 0.042 mmol) and copper(II) acetate monohydrate (1 equiv, 8.4 mg) and 1 mL of water were placed in a microwave vial (10 ml) and submerged into the ultrasound bath (1 cm depth). After 30 min, the reaction crude was purified through exclusion chromatography (Sephadex -G10) using water as eluent. After water evaporation the Cu(II) 5,10,15,20-*tetrakis*-(*N*-methyl-4-pyridinyl)porphyrinate was obtained in 53% yield.

2.1.4. Synthesis of copper (II) 5,10,15,20-*tetrakis*-(3-hydroxyphenyl)porphyrinate under ultrasound
A mixture of 5,10,15,20-*tetrakis*-(3-hydroxyphenyl)porphyrin (50 mg, 0.042 mmol) and copper(II) acetate monohydrate (1 equiv, 8.4 mg) and 1 mL of NaOH (2 M) were placed in a microwave vial (10 ml) and submerged into the ultrasound bath (1 cm depth). After 30 min, the reaction crude was neutralized with HCl (1 M) and purified liquid-liquid extraction with ethyl acetate. After dried with Na_2SO_4 and solvent evaporation the Cu(II) 5,10,15,20-*tetrakis*-(3-hydroxyphenyl)porphyrinate was obtained in 45% yield.

Table S1. Data for Atom Economy calculation

reactant	MW (g.mol-1)	Product	MW
TMePyP	1186.44	Cu(TMePyP)	1247.97
3-OHTPP	678.73	Cu(3-OHTPP)	740.26
$Cu(OAc)_2.H_2O$	199.65		

Table S2. Data for E-factor calculation

Reactant/solvents	MW (g.mol-1)	Density (g/mL)	Product	MW
TMePyP	1186.44	--	Cu(TMePyP)	1247.97
3-OHTPP	678.73	--	Cu(3-OHTPP)	740.26
$Cu(OAc)_2.H_2O$	199.65	--		
water	18.02	1		
NaOH	40.00			

Table S3. E-factor for the synthesis of Cu(II) complexes of TMePyP

Mechanochemistry				E-factor = 4.7
Reactant/solvents	m (mg)	Product	m (mg)	
TMePyP	50	Cu(TMePyP)	23.6	
$Cu(OAc)_2.H_2O$	84.1			
Ultrasound				E-factor = 37.0
TMePyP	50	Cu(TMePyP)	27.8	
$Cu(OAc)_2.2H_2O$	8.4			
H_2O	1000			

Table S4. E-factor for the synthesis of Cu(II) complexes of 3-OHTPP

Mechanochemistry				E-factor = 2.1
Reactant/solvents	m (mg)	Product	m (mg)	
3-OHTPP	50	Cu(3-OHTPP)	40.5	
$Cu(OAc)_2.H_2O$	73.8			
Ultrasound				E-factor = 27.3
3-OHTPP	50	Cu(3-OHTPP)	40.5	
$Cu(OAc)_2.H_2O$	14.7			
H_2O	1000			
NaOH	80			

Table S5. Data for Ecoscale calculation

	Cu(TMePyP) Mechanochemistry	Cu(TMePyP) Ultrasound	Cu(3-OHTPP) Mechanochemistry	Cu(3-OHTPP) Ultrasound
Parameter	Penalty points			
1.Yield	27.5	23.5	13	13
2. Price of components	5	5	5	5
3. Safety	5	5	5	10
4. Technical Setup	2	2	2	2
5. Temperature/time	0	0	0	0
6. Workup/Purification	10	10	3	3
Total penalty points	49.5	45.5	28	33
**Ecoscale**	**50.5**	**54.5**	**72**	**67**

References

1. Kumar, A.; Maji, S.; Dubey, P.; Abhilash, G. J.; Pandey, S.; Sarkar, S., One-pot general synthesis of metalloporphyrins. *Tetrahedron Lett.* **2007,** 48, (41), 7287-7290. DOI:10.1016/j.tetlet.2007.08.046.
2. Sousa, J. F. M.; Pina, J.; Gomes, C.; Dias, L. D.; Pereira, M. M.; Murtinho, D.; Dias, P.; Azevedo, J.; Mendes, A.; Seixas de Melo, J. S.; Pais, A. A. C. C.; Pineiro, M.; Valente, A. J. M., Transport and photophysical studies on porphyrin-containing sulfonated poly(etheretherketone) composite membranes. *Mater. Today Commun.* **2021,** 29, 102781 DOI: 10.1016/j.mtcomm.2021.102781.
3. Jain, N.; Kumar, A.; Chauhan, S. M. S., Synthesis of Transition Metal Porphyrins from Free-Base 5,10,15,20-Tetraarylporphyrins Under Microwave Irradiation in Ionic Liquids. *Synth. Commun.* **2005,** 35, (9), 1223-1230. DOI: 10.1081/scc-200054823.
4. Ralphs, K.; Zhang, C.; James, S. L., Solventless mechanochemical metallation of porphyrins. *Green Chem.* **2017,** 19, (1), 102-105. DOI: 10.1039/c6gc02420c.
5. Sankar, M.; Bhyrappa, P.; Varghese, B.; Praneeth, K. K.; Vaijayanthimala, G., Meso-tetrakis(3',5'-di-substituted-phenyl)porphyrins: structural, electrochemical redox and axial ligation properties. *J. Porphyr. Phthalocyan.* **2012,** 09, (06), 413-422. DOI: 10.1142/s1088424605000514.
6. Chen, E. X.; Qiu, M.; Zhang, Y. F.; Zhu, Y. S.; Liu, L. Y.; Sun, Y. Y.; Bu, X.; Zhang, J.; Lin, Q., Acid and Base Resistant Zirconium Polyphenolate-Metalloporphyrin Scaffolds for Efficient CO2 Photoreduction. *Adv Mater* **2018,** 30, (2), 1704388. DOI: 10.1002/adma.201704388.
7. Smith, K. M., *Porphyrins and metalloporphyrins* Elsevier Scientific Publishing Company: Amsterdam, 1975.
8. Joseph, R.; Kumar, K. G., Electrochemical sensing of acyclovir at a gold electrode modified with 2-mercaptobenzothiazole-[5,10,15,20-tetrakis-(3-methoxy-4-hydroxyphenyl)porphyrin ato]copper(II). *Anal Sci* **2011,** 27, (1), 67-72. DOI: 10.2116/analsci.27.67.
9. Wyrębek, P.; Ostrowski, S., Synthesis of some β-nitro-meso-tetraphenylporphyrin derivatives. *Journal of Porphyrins and Phthalocyanines* **2012,** 11, (11), 822-828, 10.1142/s1088424607000941.

10. Pasternack, R. F.; Francesconi, L.; Raff, D.; Spiro, E., Aggregation of Nickel(II), Copper(II), and Zinc(I1) Derivatives of Water-Soluble Porphyrins. *Inorg. Chem.* **1973,** 12, (11), 2606-2611.DOI: 10.1021/ic50129a023
11. Casas, C.; Lacey, C. J.; Meunier, B., Preparation of hybrid "DNA cleaver-oligonucleotide" molecules based on a metallotris(methylpyridiniumyl)porphyrin motif. *Bioconjug Chem* **1993,** 4, (5), 366-71. DOI: 10.1021/bc00023a011.
12. Pasternack, R. F.; Spiro, E. G.; Teach, M., Solution properties of nickel(II) tetra(4-N-methylpyridyl) porphine and the influence of acetone, pyridine and imidazole. *Journal of Inorganic and Nuclear Chemistry* **1974,** 36, (3), 599-606. DOI: 10.1016/0022-1902(74)80120-x.
13. Manna, B. K.; Sen, D.; Bera, S. C.; Rohatgi-Mukherjee, K. K., Spectral and flash kinetic studies of water-soluble zinc porphyrins in poly-N-vinyl-2-pyrrolidone matrix. *Spectrochimica Acta Part A: Molecular Spectroscopy* **1992,** 48, (11-12), 1657-1669. DOI: 10.1016/0584-8539(92)80239-s.
